# Genome-wide association studies reveal that members of bHLH subfamily 16 share a conserved function in regulating flag leaf angle in rice *(Oryza sativa)*

**DOI:** 10.1371/journal.pgen.1007323

**Published:** 2018-04-04

**Authors:** Haijiao Dong, Hu Zhao, Shuangle Li, Zhongmin Han, Gang Hu, Chang Liu, Gaiyu Yang, Gongwei Wang, Weibo Xie, Yongzhong Xing

**Affiliations:** 1 National Key Laboratory of Crop Genetic Improvement and National Center of Plant Gene Research (Wuhan), Huazhong Agricultural University, Wuhan, China; 2 Hubei Collaborative Innovation Center for Grain Industry, Yangtze University, Jingzhou, China; Washington University, UNITED STATES

## Abstract

As a major component of ideal plant architecture, leaf angle especially flag leaf angle (FLA) makes a large contribution to grain yield in rice. We utilized a worldwide germplasm collection to elucidate the genetic basis of FLA that would be helpful for molecular design breeding in rice. Genome-wide association studies (GWAS) identified a total of 40 and 32 QTLs for FLA in Wuhan and Hainan, respectively. Eight QTLs were commonly detected in both conditions. Of these, 2 and 3 QTLs were identified in the *indica* and *japonica* subpopulations, respectively. In addition, the candidates of 5 FLA QTLs were verified by haplotype-level association analysis. These results indicate diverse genetic bases for FLA between the *indica* and *japonica* subpopulations. Three candidates, *OsbHLH153*, *OsbHLH173* and *OsbHLH174*, quickly responded to BR and IAA involved in plant architecture except for *OsbHLH173*, whose expression level was too low to be detected; their overexpression in plants increased rice leaf angle. Together with previous studies, it was concluded that all 6 members in bHLH subfamily 16 had the conserved function in regulating FLA in rice. A comparison with our previous GWAS for tiller angle (TA) showed only one QTL had pleiotropic effects on FLA and TA, which explained low similarity of the genetic basis between FLA and TA. An ideal plant architecture is expected to be efficiently developed by combining favorable alleles for FLA from *indica* with favorable alleles for TA from *japonica* by inter-subspecies hybridization.

## Author summary

Rice leaf angle is a major component of ideal plant architecture that determines plant density. Many leaf angle-related genes have been characterized based on mutants, but natural variations of these genes and potential values in genetic improvement have not been evaluated. Here we explore the genetic basis of rice FLA in rice by GWAS. Dozens of quantitative trait loci (QTLs) have been identified, but few FLA QTLs have been commonly detected between *indica* and *japonica* subpopulations. Alleles for small leaf angle mostly come from *indica* rice. Three novel OsbHLH genes have been confirmed to regulate leaf angle by overexpression. Together with previous studies, all 6 members of bHLH subfamily 16 have a conserved function in regulating leaf angle. Only one QTL has been identified with pleiotropic effects on FLA and TA in the same collection, indicated the diverse genetic basis between FLA and TA. Alleles for small TA mostly come from *japonica* rice. We suggest to breed compact plant type cultivars by simultaneously utilizing favorable alleles for FLA and TA via inter-subspecies hybridization.

## Introduction

Leaf angle is the inclination between leaf and stem, which is an important agronomic trait that attracts attention. Erect leaves maximize carbon gain by optimizing the interception of photosynthetically active radiation for canopy photosynthesis and by mitigating heat stress induced by excess infrared radiation [1–3]. Crops with erect leaves can be grown in an increasing plant density without compensation by the photosynthesis rate, which consequently increases grain yield. Therefore, leaf erectness as one of the components of ideal plant architecture has been a breeding target for several decades [4–7]. In addition, the more upright leaves also improve the accumulation of leaf nitrogen for grain filling in rice [8].

In addition to breeders, scientists in plant developmental biology have paid much attention to the mechanism of leaf angle formation. The molecular mechanisms in leaf angle have differed among various reports, but there is a common opinion that phytohormone synergism is the key regulator of leaf angle. Endogenous hormones, especially brassinosteroids (BRs), play important roles in controlling rice leaf angle by promoting the growth of cells on the adaxial side of the lamina joint [9, 10]. Most of the rice leaf angle-related genes with regard to BR biosynthesis and BR signaling or that are otherwise BR related have been identified, such as *OsDWARF4*, *D2*/*CYP90D2*, *OsBRI1* and *OsBZR1* [11–14]. *OsIAA1* and *OsARF19* control the leaf angle by responding to auxin and BR hormones [15, 16]. *OsSPY* and *D1*/*RGA1* are two genes in the GA signaling pathway that regulate leaf angle in a BR-GA crosstalk manner in rice [17, 18]. *Increased Leaf Angle 1* (*ILA1*), different from the abovementioned leaf angle-related genes, regulates mechanical tissue formation in the rice leaf lamina joint [19]. *CYCU4;1* promotes the proliferation of sclerenchyma cells on the abaxial side of the lamina joints to affect rice leaf erectness through the BR signaling pathway [20]. *oslg1* is the T-DNA insertion mutant of *OsLIGULELESS1* (*OsLG1*), with erect leaves from the loss of the lamina joint structure [21]. Therefore, there are many findings on the development of leaf angle of rice that are helpful for understanding its molecular mechanism.

However, many of these studies are based on the reverse genetic approach. While understanding the natural variation in rice leaf angle is still limited, which is more important for breeding rice varieties with ideal plant architecture. Since the 1990s, researchers have explored several leaf angle-related quantitative trait loci (QTLs) with bi-parental mapping populations [22–24]. Recently, genome-wide association study (GWAS) has become a popular approach for QTL mapping in crops due to its strong power and high-resolution mapping [25, 26]. In maize, GWAS demonstrated that the genetic architecture of the leaf traits, including leaf angle, is dominated by multiple minor QTLs, with little epitasis or environmental interaction [27]. GWAS focused on plant architecture traits, including FLA, were conducted in two separate *indica* rice collections [28, 29]. Although several novel FLA-related loci have been detected, this alone is not comprehensive for understanding the natural variation in leaf angle in cultivated rice.

The upper canopy of the rice plant, especially the flag leaf, the top leaf after heading, intercepts most of the solar radiation at stages of heading and grain filling. FLA is closely associated with the efficiency of solar utilization by the flag leaf. It is of significance to further dissect the genetic basis of FLA for improvement of plant architecture. In this study, we performed GWAS for FLA with 529 *Oryza sativa* accessions at the heading stage using a linear mixed model (LMM). Several genome regions were associated with FLA, and 3 previously uncharacterized genes of basic helix-loop-helix (bHLH) transcriptional factor subfamily 16 were in or around the associated regions. Overexpression transgenic plant analysis confirmed that members of bHLH subfamily 16 have a conserved function in controlling rice leaf angle. Although both TA and FLA are the major components of plant architecture, a low correlation between TA and FLA and only one QTL with pleiotropic effects on both traits indicated their different genetic bases.

## Results

### Phenotypic variation of FLA at heading stage

**Fig 1:**
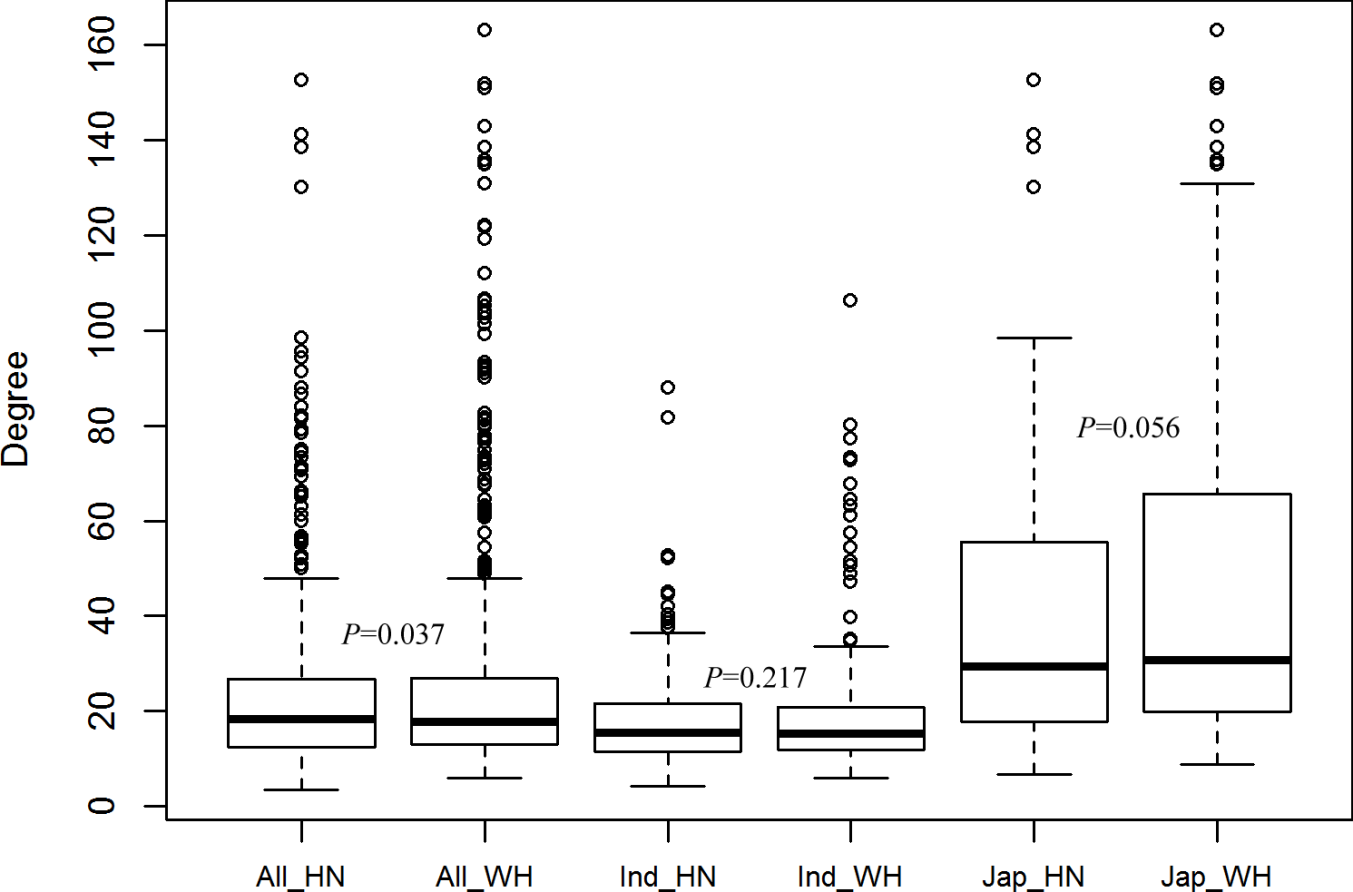
Phenotypic distribution of flag leaf angle in the germplasm collection in two environments. The boxplot shows the differences in FLA among the whole population and *indica* and *japonica* subpopulations. The box edges represent the upper and lower quantile, with the median value shown by the bold line in the middle of the box. Whiskers represent the data from the lowest quantile to the top quantile. Individuals falling outside the range of the whiskers are shown as open dots. All, Ind and Jap represent the whole population and *indica* and *japonica* subpopulations, respectively; HN and WH represent the Hainan and Wuhan environments, respectively.

The FLA of 529 *O*. *sativa* accessions shared a similar distribution in Hainan and Wuhan, China (Fig 1). There was a large variation in FLA in the whole population: from 3.5° to 152.5° in Hainan and from 6° to 163° in Wuhan. However, the FLA of one half of the accessions in the middle ranged from 15° to 30°, and the median value of the FLA was approximately 20°. FLA showed a high heritability of 0.79. FLA in the *indica* accessions had smaller variations with smaller mean values than those in *japonica* in both environments (Fig 1). The correlation coefficients of FLA between Hainan and Wuhan were 0.40 and 0.60 within the *indica* and *japonica* subpopulations that both reached a significant level (*P*<0.05), respectively. Two-way ANOVA revealed that FLA was dominantly controlled by genetic factors but also influenced by genotype-by-environment interactions (Table 1). In the *indica* subpopulation, the interaction between genotype and environment accounted for 28.4% of the variation (Table 1). We also measured TA at the heading stage for the collection grown in the two environments [30]. A significant positive correlation was observed between FLA and TA only in the *indica* subpopulation, but the correlation coefficients were small in both environments (Hainan, 0.20; Wuhan, 0.34).

**Table 1:** Summary of variances resolved by two-way analysis of variance for flag leaf angle in two environments.

SOV	Full population				*Indica* subpopulation				*Japonica* subpopulation			
	df	F	P	SSE/SST (%)	df	F	P	SSE/SST (%)	df	F	P	SSE/SST (%)
G	500	32.8	<0.0001	77.7	288	14.9	<0.0001	62.9	140	29.2	<0.0001	73.7
E	1	90	<0.0001	0.4	1	18.8	<0.0001	0.3	1	67.4	<0.0001	1.2
G×E	500	7.2	<0.0001	17.1	288	6.7	<0.0001	28.4	140	7.9	<0.0001	20
Error	1002			4.8	578			8.5	282			5.1

SOV, source of variance; G, genotype; E, environment; G×E, the interaction between genotype and environment. SSE, effect sum-of-squares; SST, total sum-of-squares.

### QTLs for FLA identified by GWAS

GWAS for FLA were performed using LMM approach in the whole population and in the *indica* and *japonica* subpopulations, respectively (S1 Fig). We detected a total of 62 QTLs in Hainan and Wuhan (Table 2; S1 and S2 Tables). They were unevenly distributed on 12 chromosomes. Chromosome 8 harbored the highest number, with 10 QTLs. Eight QTLs were commonly detected in both environments (Table 2). Of these, 3 QTLs (*qFLA1e*, *qFLA8f* and *qFLA12a*) were identified in the full population, 2 QTLs (*qFLA3a* and *qFLA11a*) were found in the *indica* subpopulations, and 3 QTLs (*qFLA3e*, *qFLA6b* and *qFLA7e*) were detected in the *japonica* populations. Additionally, *qFLA6b* and *qFLA7e* were also detected in the full population separately in Hainan and Wuhan (S1 and S2 Tables). *qFLA1g* was identified in the full population in Hainan, as well as in the *indica* subpopulation in Wuhan, while *qFLA5b* was detected both in the *indica* subpopulation in Hainan and in the full population in Wuhan (S1 and S2 Tables). We detected 22 QTLs that were unique in Hainan (S1 Table): 16, 3 and 2 QTLs were only detected in the full population, *indica* and *japonica* subpopulations, respectively, and one QTL, *qFLA1c*, was identified both in the full population and the *japonica* population. Thirty QTLs were only detected in Wuhan (S2 Table): 18, 10 and 1 QTLs were found in the full population, *indica* and *japonica* subpopulations, respectively, and one QTL, *qFLA8e*, was detected both in the full population and the *japonica* population.

**Table 2:** Significant association loci for rice flag leaf angle detected in both Hainan and Wuhan using the linear mixed model in the germplasm collection.

QTL ID	Pop	Chr	Local LD region (bp)	2013 Hainan			2014 Wuhan			Known QTLs
				SNP ID ^a^	P_LMM	Var %	SNP ID ^a^	P_LMM	Var %	
*qFLA1e*	All	1	30,417,429~31,555,541	sf0131340771	1.2E-09	0.2	sf0131304747	6.4E-10	1.6	*QLa1* [23]
*qFLA8f*	All	8	20,968,463~21,468,267	sf0821186426	8.1E-10	0.5	sf0821201486	1.8E-07	0.1	
*qFLA12a*	All	12	1,426,738~1,622,694	sf1201555804	3.6E-09	0.2	sf1201604090	3.7E-10	0.9	
*qFLA3a*	*Ind*	3	3,241,595~3,312,644	sf0303244440	6.6E-09	15.2	sf0303246326	1.4E-07	7.2	
*qFLA11a*	*Ind*	11	6,222,383~6,537,377	sf1106484837	2.1E-07	4.6	sf1106421222	8.7E-08	0.1	
*qFLA3e*	*Jap*	3	21,294,778~22,460,027	sf0322402523	1.9E-08	11.5	sf0322428486	6.0E-07	26.5	
*qFLA6b*	*Jap*	6	4,541,644~4,833,334	sf0604579127	2.1E-07	3.7	sf0604579127	8.0E-07	12.8	*QFla6* [23]
*qFLA7e*	*Jap*	7	28,893,769~29,673,928	sf0728932621	6.3E-08	2.2	sf0728932621	7.5 E-07	0.6	

a. The SNP ID is composed of three parts: sf, the number of chromosome and the genome position (MSU.V6), eg. sf0105241388 indicates the SNP located in 5,241,388bp on chromosome 1 (MSU.V6).

### Co-localization of associated sites with previously reported QTLs/genes for FLA

We compared the genomic positions of known leaf angle genes with the associated sites detected in this study. Four genes were co-localized with our associated sites (S1 and S2 Tables): *OsBRI1*, an orthologue of Arabidopsis *BRI1*, which plays an important role in BR signaling [14], was located in the linkage disequilibrium (LD) region where *qFLA1d* was detected in Wuhan. *PGL1* and *PGL2*/*OsBUL1*, the homologs of *OsILI1* and *BU1* in rice [31–33], were located in the LD regions of *qFLA3b* detected in Hainan and *qFLA2f* detected in Wuhan, respectively. *OsSPY*, encoding an O-linked N-acetylglucosamine transferase [17], was located in the region of *qFLA8j* detected in Wuhan.

In addition, we compared the localization of the associated sites detected in this study with previously detected leaf angle QTLs with bi-parental mapping populations from the gramene website (http://www.gramene.org) and with the significant FLA loci detected via GWAS in previous studies [28, 29]. A total of 11 associated sites were co-localized with 8 previously reported QTLs (Table 2; S1 and S2 Tables): both *qFLA1e* and *qFLA6b*, commonly identified in both environments, were located in the regions of *QLa1* and *QFla6*, respectively; *qFLA6c* and *qFLA6d*, detected only in Wuhan, were located in the region of *QFla6*; *qFLA9c* and *qFLA9d*, only identified in Hainan, were co-localized with *fla9*; *qFLA5b* was located in the *QFla5* region; and *qFLA1f*, *qFLA2f*, *qFLA3d* and *qFLA7d*, only detected in Wuhan, were also located in the previous QTL regions. These results indicated the reliability of FLA-related associations in our study.

### Haplotype-level association analysis of genes of bHLH subfamily 16

Three bHLH genes, such as *OsILI1* (*OsbHLH154*), *BU1* (*OsbHLH172*) and *PGL2*/*OsBUL1* (*OsbHLH170*), control leaf angle and belong to bHLH subfamily 16 [32–37]. Interestingly, *OsbHLH173* and *OsbHLH174*, two members of subfamily 16, are in the local LD regions where the lead SNP of *qFLA10c* was located (S1 Table). In addition, *OsbHLH153* was located close to the QTL *qFLA3b* (S1 Table). They are likely the genes underlying these QTLs. Haplotype level association analysis frequently improves the power of QTL mapping [26, 38, 39]. To provide more evidence for their identities, we constructed the haplotypes of all 6 members of bHLH subfamily 16 and tested the difference in FLA between all possible haplotype pairs. All these genes except *BU1* were highly significantly associated with FLA in the whole population in both environments (S3 Table). *OsbHLH153*, *OsbHLH174* and *ILI1* were significantly associated in the *japonica* subpopulation in Hainan, but none of the 6 genes were associated in the *indica* subpopulation (S3 Table).

**Fig 2:**
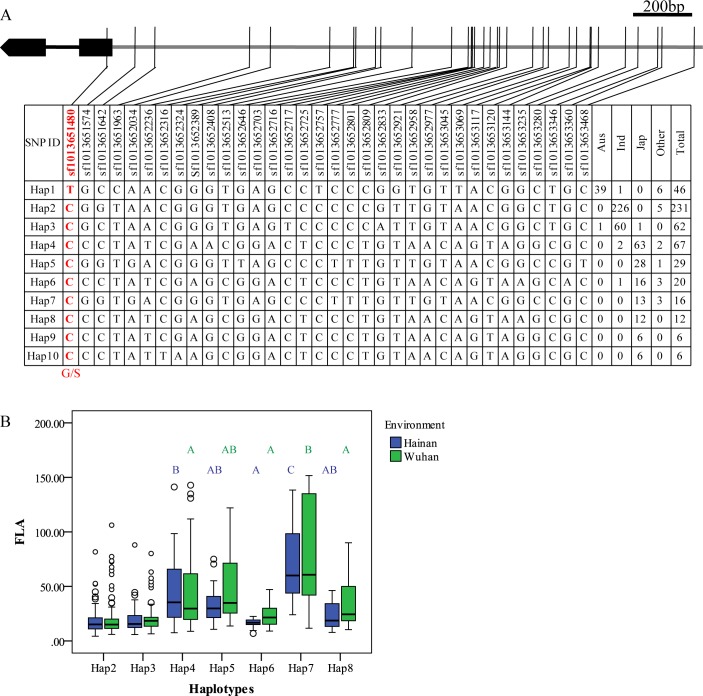
Haplotype analysis of *qFLA10c*/*OsbHLH174*. (A) Major haplotypes (haplotypes each carried by more than 5 accessions) of *OsbHLH174* in the whole population according to SNPs data from RiceVarMap version 1. The region contains 2 kb upstream and coding region. The SNP in red and bold is a non-Synonymous SNP. (B) Comparison of FLA between Hap2 and Hap3 in *indica* rice and FLA among Hap4-Hap8 in *japonica* rice using an independent *t*-test and a Duncan’s test (*P*< 0.01), respectively.

Here we presented the results of *OsbHLH174* and *OsbHLH173* as the candidate genes of *qFLA10c* and *OsbHLH153* as the candidate gene of *qFLA3b*. Only one SNP (sf1013651480) causing amino acid change (S8G) was detected in *OsbHLH174* coding region (Fig 2A). Most *aus* accessions belong to Hap1 containing Ser8, while most *indica* and *japonica* accessions were divided into Hap2-Hap10 shared Gly8 (Fig 2A). The effects of two major haplotypes in the *indica* subpopulation were similar, while the effects were significantly different among major haplotypes in the *japonica* accessions, especially between Hap6 and Hap7 (Fig 2B). Ser and Gly are both uncharged hydrophilic amino acids, and both have similar biochemical characters. Therefore, sf1013651480 was not a functional nucleotide polymorphism site (FNP), and the nucleotide change in promoter may lead to the significant difference for FLA. Accordingly, only a SNP (sf1013702588) causing a non-synonymous mutation (S3G) was detected in *OsbHLH173*, and most *aus* and *indica* accessions carried Ser at this site (S2A Fig). Although significant differences in FLA were detected among haplotypes in *japonica* (S2B Fig), sf1013702588 was not associated with leaf angle. This result suggested the genetic variation of promoter controlling FLA. For *OsbHLH153*, one SNP (sf0303844743) caused a non-synonymous mutation (G12V) (S3A Fig). Most *aus*, *indica* and *japonica* accessions carried Gly at site 12 (Haplotypes 1–5 and 7). A small proportion of *japonica* accessions carried Val at site 12 (haplotypes 6 and 8). Unlike Gly, Val is an uncharged hydrophobic amino acid. Within *japonica* rice, FLA of Hap5 with G12 was significantly smaller than that of Hap6 with V12 in Hainan (S3B Fig). In addition, some SNPs in the promoter region were also associated with FLA such as sf0303844574, sf0303844528, sf0303844032, sf0303843859 and sf0303843700 (S3A Fig). Therefore, both polymorphisms in coding and promotor regions of *OsbHLH153* caused the variation of leaf angle. To verify the contribution of promotor variation, we firstly examined the expression pattern of these bHLH genes with the rice chip DB “CREP” (http://crep.ncpgr.cn/crep-cgi/home.pl), and found they preferably expressed in young and growing tissues but not in mature tissues (S4B Fig). Then we sampled the flag leaf from 296 accessions for RNA sequencing. Accordingly, it was hard to detect the expressions of all the six members of bHLH subfamily 16 (S6 Table). Therefore, it was failed to perform the associations between expression amounts of these three bHLH genes and FLA.

### Overexpression of *OsbHLH153*, *OsbHLH173* and *OsbHLH174* increased rice leaf angle

**Fig 3:**
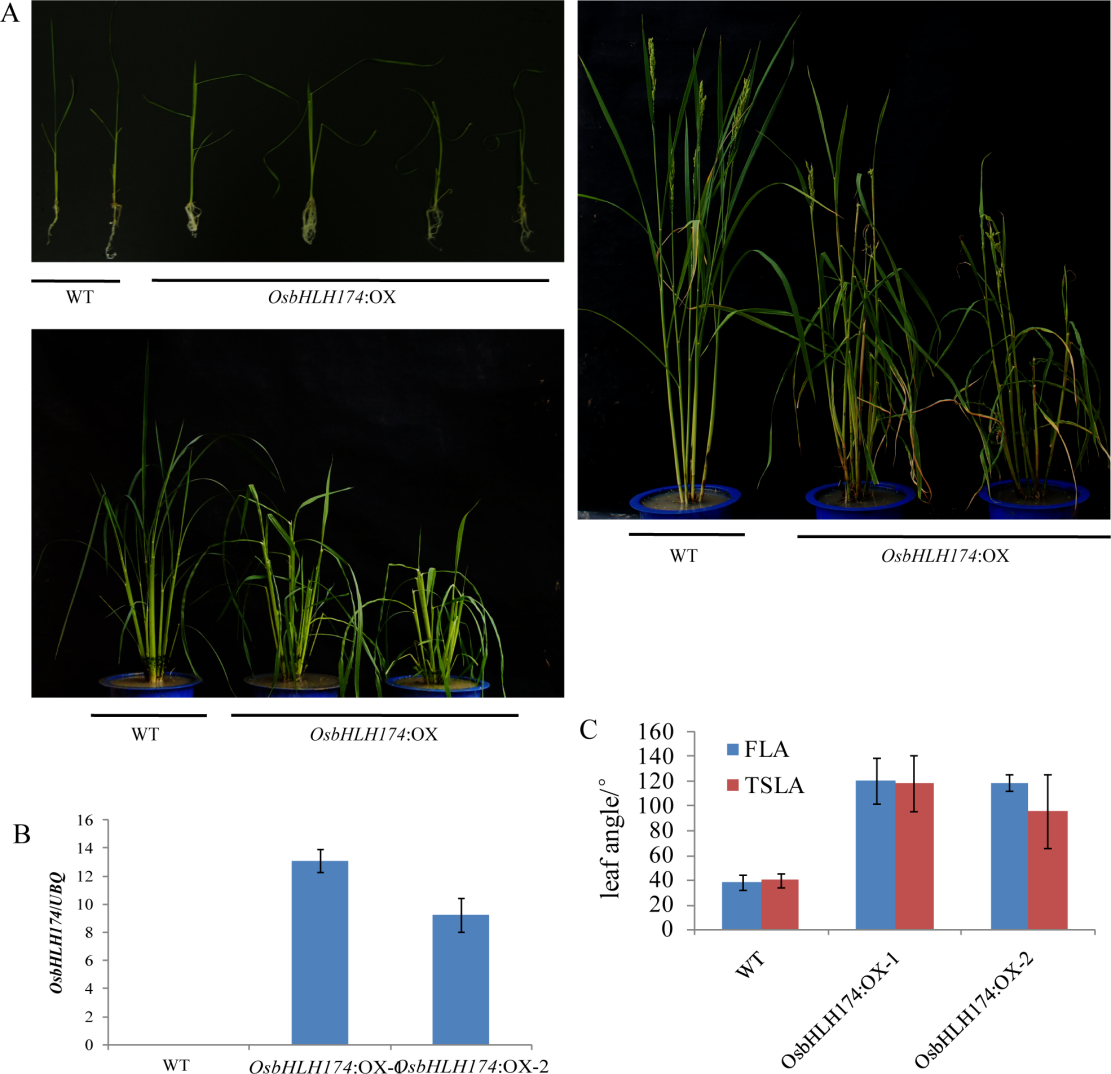
*OsbHLH174* overexpression transgenic plants showed an increase in the leaf angle. (A) The morphology of wild type (WT) and *OsbHLH174*: OX plants, at the seedling, tillering and heading stages. (B) quantitative real-time reverse transcription-polymerase chain reaction (qRT-PCR) analysis of *OsbHLH174* transcripts in WT and *OsbHLH174*: OX at the seedling stage. (C) FLA and TSLA of the wild type and *OsbHLH174*:OX-1 and -2 at the heading stage (*P*< 0.001, n≥ 5).

To test whether the expression level of these genes affects leaf angle, then we generated overexpression plants for these 3 bHLH genes. Many *OsbHLH174* overexpression plants (T0) showed a significantly increased leaf angle (Fig 3A). We measured FLA and the top second leaf angle (TSLA) in two T1 overexpressing lines. All the overexpression plants showed significantly increased lamina joint bending (Fig 3B and 3C). T0 overexpression plants of *OsbHLH153* and *OsbHLH173* showed an increased leaf angle, and some of these exhibited defective phenotypic variations, including leaf and stem twisting (S5 Fig). A mutation in the promoter of *Style2*.*1*, a homolog of these rice bHLHs genes, resulted its decreased expression and differentiated its function in cultivated tomatoes [40]. We overexpressed *Style2*.*1* from tomato *Solanum pennellii* in *japonica* rice variety Zhonghua 11. The overexpression plants showed an increased lamina joint (S6 Fig).

### Responses of *OsbHLH* genes to exogenous phytohormones

**Fig 4:**
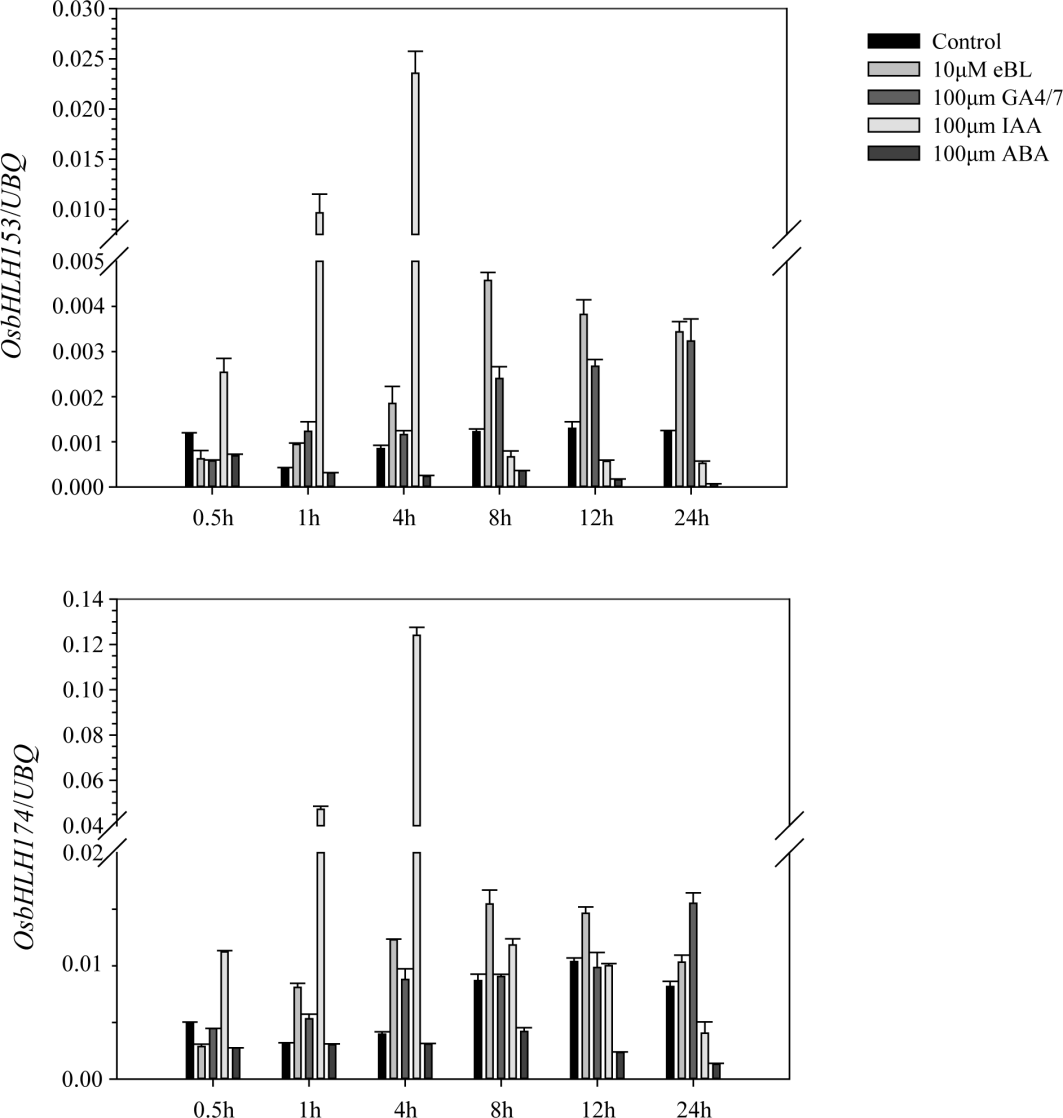
Expression pattern of *OsbHLH153* and *OsbHLH174* in response to exogenous IAA, eBL, GA and ABA. qRT-PCR analysis of the transcripts of *OsbHLH153* and *OsbHLH174* in Zhonghua 11 wild type treated with 100 μM IAA, 10 μM eBL, 100 μM ABA and 100 μM GA as the experimental groups and with water as the control group for different times. Data are the means ± SD (n = 3).

As many leaf angle-related genes are regulated by plant hormones, especially BRs, we treated the three-leaf seedlings with 4 kinds of hormones and checked the expression change of these 3 bHLH genes, *OsbHLH153*, *OsbHLH173* and *OsbHLH174*, by quantitative real-time reverse transcription-polymerase chain reaction (qRT-PCR). *OsbHLH153* and *OsbHLH174* were up-regulated immediately and reached a maximum level of over 27× compared with the control group at 4 h after treatment with 100 μM indole-3-acetic acid (IAA), whereas they gradually decreased expression after treatment with 100 μM abscisic acid (ABA) (Fig 4). The expression of *OsbHLH153* was up-regulated to a maximum level (3.7×) at 8 h after treatment with 10 μM epibrassinolide (eBL) and increased 3.0× at 1 h after treatment with 100 μM GA4/7 compared with the control group (Fig 4). The expression of *OsbHLH174* reached 3.1× and 2.2× at 4 h when treated with 10 μM eBL or 100 μM GA4/7, respectively, compared with the control group (Fig 4). However, the expression of *OsbHLH173* was too low to be detected in seedling and other tissues (S4A Fig). These results suggested that these bHLH genes were regulated by plant hormones and might regulate leaf angle by these hormone pathways.

### Haplotype effects of *OsBRI1* on FLA

**Fig 5:**
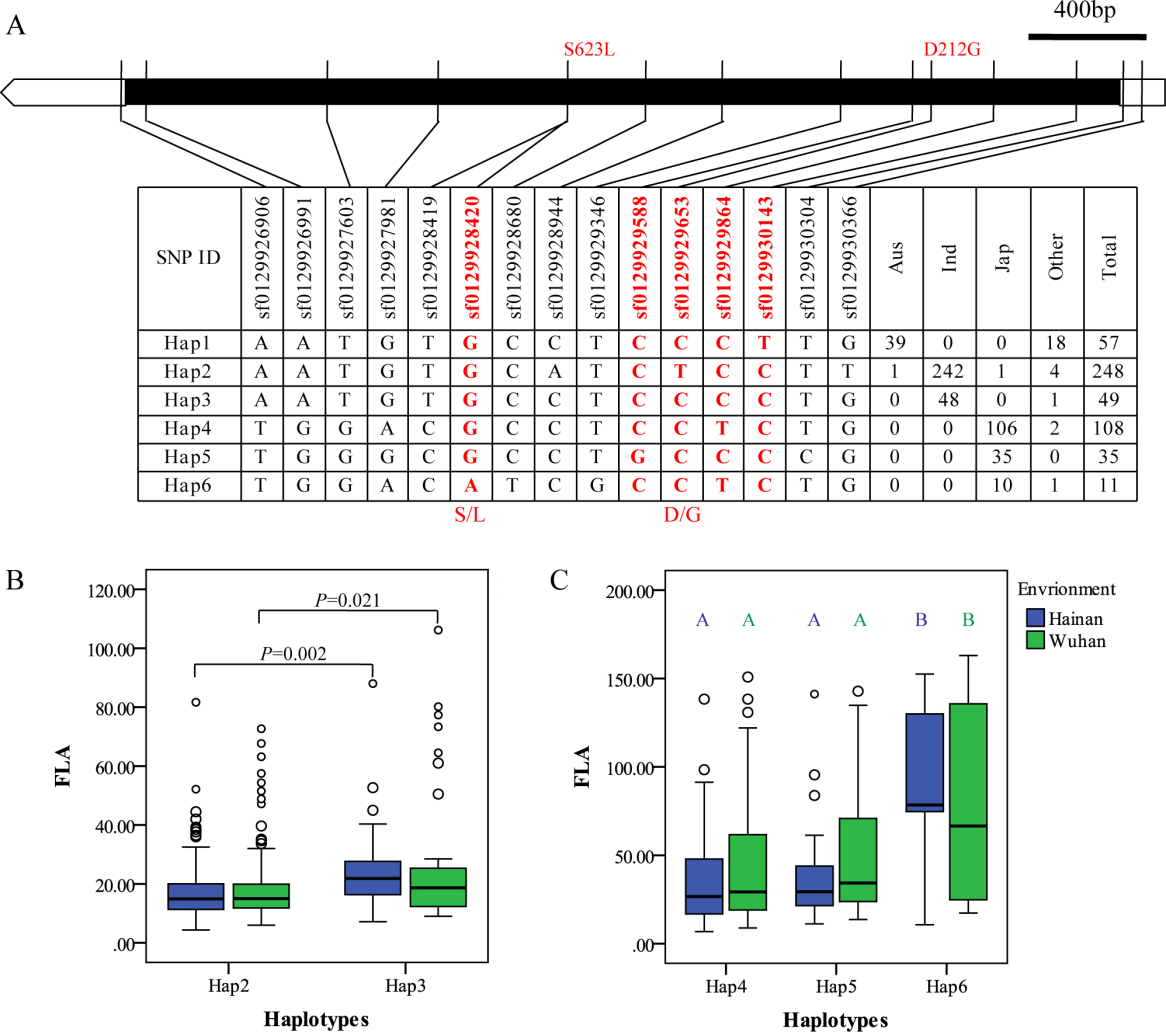
Haplotype analysis of *qFLA1d*/*OsBRI1*. (A) Major haplotypes (haplotypes each carried by more than 5accessions) of *OsBRI1* in the full population according to SNPs data from RiceVarMap version 1. The SNPs in red and bold are non-Synonymous SNPs. (B) Comparison of FLA between Hap2 and Hap3 in *indica* rice by an independent *t*-test. (C) Comparison of FLA among Hap4-Hap6 in *japonica* rice by a Duncan’s test (*P*< 0.01), respectively.

*qFLA1d*/*OsBRI1* made a large contribution to FLA variation in the full population in Wuhan (S2 Table). Haplotype-level association analysis showed that *OsBRI1* was strongly associated not only in the whole population in two environments but also in the *indica* and *japonica* subpopulations in Hainan (S3 Table). A total of 6 major haplotypes were constructed based on all SNPs in *OsBRI1*. Most *indica* accessions belong to Hap2 or Hap3, and most *japonica* rice carried Hap4-Hap6 (Fig 5A). Within *indica* subpopulation, the flag leaf of accessions carried Hap2 (D212; Asp, an amino acid with a negative charge of polarity) was more erect than those carried Hap3 (G212; Gly) (Fig 5A and 5B). Within *japonica*, FLA of the accessions carried OsBRI1-Hap6 (L623; Leu, a nonpolar amino acid) was larger than those carried OsBRI1-Hap4 (S623; Ser, an uncharged hydrophilic amino acid) and OsBRI1-Hap5 (S623) (Fig 5A and 5C). SNPs sf0129929653 and sf0129928420 might be FNPs separately controlling leaf angle in the *indica* and *japonica* subpopulations.

### Large effects of gene combinations between *OsbHLHs* and *OsBRI1*

**Fig 6:**
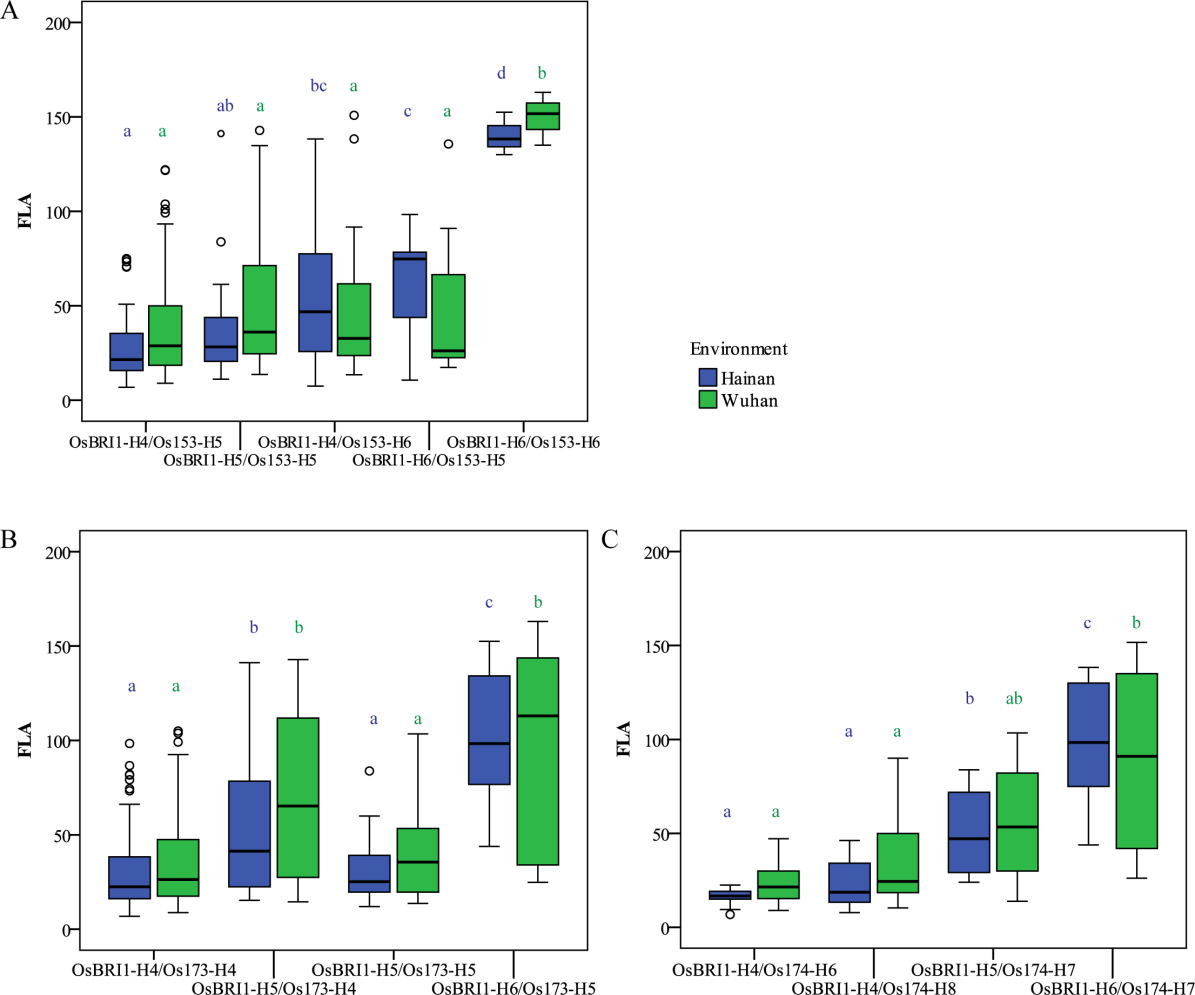
The genetic interactions between *OsbHLHs* and *OsBRI1* in *japonica*. Comparison of the FLA among the combinations of haplotypes of *OsBRI1* with three *OsbHLHs*, *OsbHLH153* (A), *OsbHLH173* (B) and *OsbHLH174* (C), using boxplots by a Duncan’s test (*P*< 0.05) in *japonica*, respectively.

Three members of OsbHLH 16 subfamily genes acted downstream of *OsBRI1* in BR signaling pathway [33, 36, 37]. Based on the hypothesis that *OsBRI1* combined with its downstream genes controlling leaf angle with varied effects. Then we investigated effects of gene combinations between three *OsbHLHs* identified in this study and *OsBRI1* on FLA in *japonica* subpopulation (Fig 6). Different combinations showed significant differences in FLA. The combinations constructed by haplotypes with small FLA such as OsBRI1-Hap4/OsbHLH153-Hap5, OsBRI1-Hap5/OsbHLH153-Hap5, OsBRI1-Hap4/OsbHLH173-Hap4, OsBRI1-Hap4/OsbHLH174-Hap6 and OsBRI1-Hap4/OsbHLH174-Hap8 had small FLA; and the combinations constructed by haplotypes with large FLA such as OsBRI1-Hap6/OsbHLH153-Hap6, OsBRI1-Hap6/OsbHLH173-Hap5 and OsBRI1-Hap6/OsbHLH174-Hap7 had large FLA. The other types usually had intermediate FLA such as OsBRI1-Hap5/OsbHLH174-Hap7. These results indicated that selection for the combinations between these three *OsbHLHs* and *OsBRI1* should be more important and efficient than for single gene when improving leaf angle in *japonica* subpopulation.

## Discussion

### Diverse genetic bases of FLA between *indica* and *japonica* subpopulations

In this study, we found that *indica* rice has a smaller FLA with a narrower distribution than *japonica* rice in Hainan and Wuhan (Fig 1). A total of 17 and 8 unique associations were detected by GWAS in the *indica* and *japonica* subpopulations, respectively. However, only 2 and 3 were commonly detected in both environments in *indica* and *japonica* subpopulations, respectively (Table 2; S1 and S2 Tables). Further haplotype-level association analysis of leaf angle-related genes located in or around the regions of associations in the two subpopulations indicated that significant differences in FLA between/among haplotypes were detected for all these genes in the *japonica* accessions in Hainan, while no significant difference was observed for these genes, except for *OsBRI1*, in the *indica* accessions (S3 Table, Figs 2 and 5; S2 and S3 Figs). So, we concluded that FLA has undergone diversifying selection. The *indica* subpopulation has been fixed with non-functionally differential haplotypes of the leaf angle-related genes, whereas the spread of functionally differentiated haplotypes in *japonica* has led to a wider variation in FLA. Therefore, there are diverse genetic bases of FLA between the two subpopulations, and some genes regulate FLA, dependent on the environment.

### The genes of bHLH subfamily 16 have a conserved function in controlling rice leaf angle

The bHLH family, a large family of transcription factors, is found throughout the eukaryotic kingdoms. The basic region functions as a DNA-binding motif, and the HLH region allows the homodimer or heterodimer formation [34, 35, 41–43]. Many *bHLHs* have been functionally characterized with multiple functions in regulating many biological developments. bHLH subfamily 16 is composed of several atypical proteins that modulate the expression of downstream genes by forming heterodimers as non-DNA-binding bHLHs. The *PREs* in Arabidopsis and *style2*.*1* in tomato, which belong to bHLH subfamily 16, have been reported to regulate cell elongation in different tissues [40, 44]. In rice, there are 6 genes in bHLH subfamily 16. Of these, *OsILI1* (*OsbHLH154*), *BU1* (*OsbHLH172*) and *PGL2*/*OsBUL1* (*OsbHLH170*) were previously confirmed to regulate leaf angle [33, 36, 37]. In this study, the remaining genes of subfamily 16, *OsbHLH153*, *OsbHLH173* and *OsbHLH174*, were associated with FLA by GWAS (S1 and S2 Tables) and by haplotype analysis (Fig 2, S2 and S3 Figs).

There were few SNPs caused non-synonymous in these three bHLH genes. Meanwhile, the mutations in promoters were associated with the variation of FLA (Fig 2, S2 and S3 Figs). However, we failed to establish the relation between the expression levels of these candidate bHLH genes and FLA because their expressions were too low to be detected in flag leaf (S4 Fig, S6 Table). It is noticed that IBH1 (*ILI1* Binding bHLH Protein 1), formed a heterodimer with ILI1 and its homologs, and its activity inhibited by ILI1, could be detected in mature organs [33, 37]. A negative correlation was detected between expression of *OsIBH1* and FLA within 64 *japonica* accessions (-0.255, *P* = 0.042) that was consistent with its negative regulation to FLA. The accessions carried haplotypes of *OsbHLH153* and *OsbHLH174* with smaller FLA had higher expression levels of *OsIBH1* which indicated the expression of *OsbHLH153* and *OsbHLH174* might be associated with FLA in *japonica* (S7 Fig). Moreover, overexpressing these three *bHLHs* increased the leaf angle, like the phenotypic change of overexpressing *OsILI1*, *BU1* and *PGL2*/*OsBUL1* (Fig 3, S5 Fig). Overexpressed tomato *Style2*.1 in rice plants also enlarged the FLA (S6 Fig). Although *OsILI1* was not associated with FLA at SNP level in this study, haplotype-level association analysis showed that it was associated with FLA in *japonica* rice (S3 Table). We conclude that the genes in bHLH subfamily 16 had a conserved function in controlling rice leaf angle together with previous studies. In addition, all the members of bHLH subfamily 16 had different expression levels in vivo, but there were similar expression patterns (S4 Fig).

### Low similarity in the genetic basis between FLA and TA

Both FLA and TA are important components of plant architecture. The erect growth of cultivated rice showed a smaller TA compared with the prostrate growth of wild rice (*O*. *rufipogon*), which was a critical domestication event. A wider phenotypic variation of FLA, from 3.3° to 166.7° (Fig 1), was observed than that of the TA, which ranged from 1.8° to 34.4° [30] in the same collection used in this study. In general, *japonica* rice has a compact plant architecture, which exhibits a small tiller angle [30]. However, this is completely the opposite for FLA. Specifically, *indica* rice has a smaller FLA than *japonica* rice. Therefore, a very low correlation efficient was detected between FLA and TA, indicating distinct genetic bases for FLA and TA. Previous studies also reported a low correlation coefficient [23, 24, 29]. Therefore, the possibility of the co-localization of QTLs for leaf angle and TA or QTLs with pleiotropic effects on both traits is limited.

In fact, there are very few QTLs with pleiotropic effects on TA and FLA in rice cultivars. It has been reported that only *Ta* on chromosome 9 and *QFla5* had pleiotropic effects on TA and FLA [23]. Here, we compared the genome regions of 62 FLA-related QTLs with 30 QTLs for TA in our previous work [30] and found that only one QTL, *qFLA8f*, detected in the full population was co-located with TA QTLs *qTA8a* and *qTA8b*, likely indicating a new pleiotropic QTL for TA and FLA or two linked genes in this region. More importantly, haplotype analysis showed that the haplotypes of 6 FLA genes were functionally differentiated in *japonica* accessions, while those in *indica* accessions were not functionally differentiated, except for *OsBRI1* (Figs 2 and 5, S2 and S3 Figs, S3 Table). However, this is almost the opposite for TA-related genes. Specifically, the haplotypes of the TA-related genes are not functionally differentiated in *japonica* accessions and are fixed with functional alleles, decreasing the TA [30]. These results suggested that these genes have no pleiotropic effects on TA and FLA. Thus, it is promising that cultivars with compact plant status may be developed by combining favorable *indica* original alleles for leaf angle and *japonica* original alleles for TA without considering linkage drag.

In summary, FLA is mainly determined by genetic factors in rice, and different genetic factors control the variation of FLA in the *indica* and *japonica* subpopulations. The members of bHLH subfamily 16 have the conserved function regulating rice FLA. There is a low correlation coefficient between FLA and TA, and very few QTLs with pleiotropic effects on both traits indicate their diverse genetic bases. The ideal plant architecture in rice may be efficiently developed by combining favorable alleles for both traits by *indica*-*japonica* hybridization.

## Materials and methods

### Plant materials and field experiments

A diverse worldwide collection consisting of 529 *O*. *sativa* landraces and elite accessions was sown at the experimental farm of Huazhong Agricultural University in the winter of 2013 in Hainan and in the 2014 rice growing season in Wuhan, China. The 2-year field experiment was designed with 2 replicates per year. The FLA of 5 plants in the middle for each accession 5 days after flowered was used for FLA measurement. The angle between flag leaf and stem was measured by a protractor. The average FLA across 2 replicates within one year was used for GWAS. The basic information of the 529 *O*. *sativa* accessions is available in the RiceVarMap (http://ricevarmap.ncpgr.cn/) [45]. The average FLAs used for GWAS are shown in S4 Table.

### Two-way analysis of variance and heritability

Two-way analyses of variance were separately used to test significant difference between environments and genotypes for the whole population and two subpopulations. The analysis was run in the program Statistica 7.0 (StatSoft. Tulsa, OK, USA). Broad-sense heritability (*H*^*2*^) of FLA in the whole population was calculated based on the experiments using the formula: $H^{2}=\delta_{g}^{2}/(\delta_{g}^{2}+\delta_{ge}^{2}/n+\delta_{e}^{2}/nr)$, where $\delta_{g}^{2}$, $\delta_{e}^{2}$ and $\delta_{ge}^{2}$ were the estimates of genetic, genotype by environment and error variances derived from the mean square expectations of two-way analysis of variance (ANOVA), respectively; n was the number of environments and r was the number of replicates.

### GWAS for FLA

The whole genomic DNA sequences of the 529 cultivar accessions were genotyped with approximately 2.5×coverage genome sequencing using a bar-coded multiplex sequencing approach on an Illumina Genome Analyzer II [46]. The diverse global rice collection was classified into 9 subpopulations: *indI*, *indII*, *indica* intermediate, *Tej*, *Trj*, *japonica* intermediate, *Aus*, *VI* and intermediate [46]. Of these 529 varieties, 295 were classified into the *indica* subpopulation, including *indI*, *indII* and *indica* intermediate, and 156 were classified into the *japonica* subpopulation, including *Tej*, *Trj* and *japonica* intermediate. To control spurious associations, population structure and kinship were regarded as cofactors when performing GWAS using LMM by the FaST-LMM program [47, 48]. Kinship was calculated as a realized relationship matrix using FaST-LMM program. Population structure was calculated as Q matrix base on the admixture model[47]. A total of 3,916,415, 2,767,159 and 1,857,845 SNPs (minor allele frequency (MAF) ≥0.05; the number of accessions with minor alleles ≥ 6) were employed for GWAS in the full population, *indica* and *japonica* subpopulations, respectively. 757,578, 571,843 and 245,348 effective independent SNPs (Me) which were calculated using a method described by Li et al [49] were found in the full population and *indica* and *japonica* subpopulations, respectively. The suggestive *P* values (1/Me, 1.3×10^−6^ for the full population, 1.8×10^−6^ for *indica* and 4.1×10^−6^ for *japonica*) were used as the thresholds for associations commonly detected in Hainan and Wuhan or detected only in one environment, but the candidate genes were in their LD regions. Genome-wide significance thresholds (0.05/Me) of 6.6×10^−8^, 8.7×10^−8^ and 2.0×10^−7^ calculated by a modified Bonferroni correction were used for the full population and the *indica* and *japonica* subpopulations, respectively, for the associations detected only in Hainan or Wuhan. To obtain independent association signals, multiple SNPs exceeding the threshold in a 5-Mb region were clustered based on an *r*^*2*^ of LD ≥ 0.25; the SNPs showing the minimum *P* value in a cluster were considered to be the lead SNPs [50].

### LD and haplotype analysis

LD was investigated based on standardized disequilibrium coefficients (*D’*) and squared allele-frequency correlations (*r*^*2*^) for the pairs of SNP loci. The extent of genome-wide LD decay in the different populations was shown in previous studies [50, 51]. The distances in LD decay in the regions surrounding the lead SNPs identified in this study were calculated, and the method was described in our previous study [30].

The SNPs of the targeted genes in the 529 *O*. *sativa* accessions were obtained from the RiceVarMap (http://ricevarmap.ncpgr.cn/) using the gene ID, while for *OsbHLH153*, *OsbHLH173* and *OsbHLH174*, the SNPs in their 2kb promoter regions for the few SNPs in the CDS coordinates were added. The haplotypes of the individual genes carried by at least 10 accessions were used for comparison. An independent *t*-test and a Duncan’s test were used to compare the differences in the FLA between/among haplotypes using the SSPE program [52]. The method of haplotype-level association analysis of candidate genes was described by our previous studies [38].

### Vector construction and rice transformation

Genomic DNA fragments of *OsbHLH153*, *OsbHLH173* and *OsbHLH174* were amplified from *Nipponbare* DNA, and a genomic DNA fragment of *Style2*.*1* was amplified from tomato (*Solanum pennellii*) DNA with gene-specific primers (S5 Table) using the high-fidelity LA Taq polymerase (Takara). The PCR products without mutations were cloned into PU1301 with a maize (*Zea mays*) *Ubiquitin* promoter or into pCAMBIA1301s with the 35S promoter. The constructs were then introduced into Zhonghua 11 (ZH11) by *Agrobacterium tumefaciens*-mediated transformation using callus induction from mature embryos as subjects [53, 54]. At least two independent overexpression plants for each construct were used for measuring the FLA.

### Identification of positive transgenic plants

The total DNA were extracted from fresh leaves using the CTAB method [55]. First, the GUS fragment was amplified from the transgenic plants with the primers GUS-F and GUS-R (S5 Table) to identify the positive transgenic plants. The positive and negative transgenic plants checked by GUS amplification were then selected to compare their expression levels of the target genes by qRT-PCR.

### Exogenous eBL, IAA, GA and ABA treatment

The wild type seeds were sown and germinated on agar medium. After two weeks, the seedlings were transferred to water. On the second day that the plants were grown in water, 4 kinds of hormones were separately added in the water, ensuring the final concentrations of 10 μM eBL, 100 μM IAA, 100 μM GA and 100 μM ABA in the experimental group, with nothing added to the water in the control group. Total RNA was extracted from the whole seedling, except for the root tissue, after treatment for 0.5, 1, 4, 8, 12 and 24 h, respectively. We then analyzed the expression pattern by qRT-PCR.

### RNA extraction and expression analysis

Total RNA was extracted using an RNA extraction kit (TRIzol reagent, Invitrogen). RNA sequencing data of *OsbHLH* genes were listed in S6 Table. The expression patterns of the FLA genes were then analyzed by qRT-PCR. Measurements were obtained using the relative quantification method. Expression levels were normalized against expression of a *ubiquitin* (*UBQ*) gene. Error bars indicate standard deviations (n = 3). All primers used for qRT-PCR are listed in S5 Table.

## Supporting information

S1 Table Significant association loci for rice flag leaf angle only detected in Hainan using the LMM. (DOCX)

S2 Table Significant association loci for rice flag leaf angle only detected in Wuhan using the LMM. (DOCX)

S3 Table Haplotype-level association analysis of candidate genes using 529 accessions. (DOCX)

S4 Table The FLA of 529 *O*. *sativa* in Hainan and Wuhan. (XLS)

S5 Table Primers used in this study. (DOCX)

S6 Table The RNA seq data of OsbHLH genes in the flag leaf of 296 of 529 *O*.*sativa*. (XLS)

S1 Fig Genome-wide association study for FLA in the whole population, the *indica* and *japonica* subpopulations by LMM. Manhattan plots and quantile-quantile plots for flag leaf angle in the full population (A), the *indica* subpopulation (B) and *japonica* subpopulation (C). The horizontal dashed lines of the Manhattan plots indicate the significance thresholds that are defined in the section of materials and methods. Lambda of quantile-quantile plots represents the expected null distribution and the observed *P* value.
(TIF)

S2 Fig The haplotype analysis of *qFLA10c*/*OsbHLH173*. (A) Major haplotyes (haplotypes with more than 5accessions) of *OsbHLH173* in the full population according to SNPs data from RiceVarMap version 1. The region contains 2-kb upstream and coding region. The SNP in red and bold is a non-Synonymous SNP. (B) Comparison of the FLA between Hap2 and Hap3 in *indica* rice and the FLA among Hap4-Hap7 in *japonica* rice using an independent *t*-test and a Duncan’s test (*P*< 0.05), respectively.
(TIF)

S3 Fig The haplotype analysis of *QFLA3b*/*OsbHLH153*. (A) Major haplotyes (haplotypes with more than 5accessions) of *OsbHLH153* in the full population according to SNPs data from RiceVarMap version 1. The region contains 2 kb upstream and coding region. The SNP in red and bold is a non-Synonymous SNP. (B) Comparison of the FLA among Hap2-Hap4 in *indica* rice and the FLA between Hap5 and Hap6 in *japonica* rice using a Duncan’s test (*P*< 0.01) and an independent *t*-test, respectively.
(TIF)

S4 Fig Expression profiles of the subfamily 16 of bHLH. (A) qRT-PCR expression analysis of *OsbHLH153*, *OsbHLH173* and *OsbHLH174* in different tissues of wild type rice. The samples of Root, Stem, Leaf blade, Leaf sheath and Leaf joint were harvested at the transition period of vegetative growth to reproductive growth. (B) The expression profiling of subfamily 16 of bHLH in different tissues, and the expression data was extracted from a database (http://crep.ncpgr.cn/crep-cgi/home.pl). The expression data of *BU1* was missing.
(TIF)

S5 Fig The phenotypes of overexpressing *OsbHLH153* and *OsbHLH173* transgenic plants. Overexpressing *OsbHLH153* (A) and *OsbHLH173* (C) transgenic plants increased leaf angle; (B) qRT-PCR analysis of the *OsbHLH153* in wild type (WT) and *OsbHLH153*: OX using leaves at seedling stage; (D) qRT-PCR analysis of the *OsbHLH173* in WT and *OsbHLH173*: OX using leaves at seedling stage.
(TIF)

S6 Fig Overexpressing *Style2*.*1* transgenic plants increased leaf angle. (A) The plant status of WT and *Style2*.*1*: OX at tillering stage; (B) FLA and TSLA of the wild type and *Style2*.*1*: OX, n≥ 5; (C) genotyping of transgenic plants *Style2*.*1*: OX; (D) reverse transcription PCR (RT-PCR) analysis of the transcripts of *Style2*.*1* in WT and *Style2*.*1*: OX at seedling stage.
(TIF)

S7 Fig Comparison of the expression levels of *IBH1* among/between haplotypes of *OsbHLH174*, *OsbHLH173* and *OsbHLH153* in *japonica* using RNA-seq data. Comparison of the expression level of *OsIBH1* among/between the haplotypes of *OsbHLH174* (A), *OsbHLH153* (C) and *OsbHLH173* (E) in *japonica* using RNA-seq data by a Ducan’s test (*P*< 0.05). Comparison of FLA among/between the haplotypes of *OsbHLH174* (B), *OsbHLH153* (D) and *OsbHLH173* (F) in the corresponding *japonica* accessions by a Ducan’s test (*P*< 0.05).
(TIF)
