# Supplementary material for: Genome-wide association studies reveal that members of bHLH subfamily 16 share a conserved function in regulating flag leaf angle in rice (Oryza sativa)
Source: PLoS Genet. 2018 Apr 4;14(4):e1007323. doi: 10.1371/journal.pgen.1007323 (PMC5902044; doi:10.1371/journal.pgen.1007323)
Supplement: S1 Table — (DOCX) [file pgen.1007323.s001.docx]

**S1 Table Significant association loci for rice flag leaf angle only detected in Hainan using the LMM**

| QTL ID | Chr | Local LD region (bp) | Class | SNP ID ^a^ | P_LMM | Var % | Known genes ^b^ | Known QTLs |
| --- | --- | --- | --- | --- | --- | --- | --- | --- |
| *qFLA1a* | 1 | 11,741,983~12,497,143 | All | sf0112429224 | 1.6E-09 | 6.5 |  |  |
| *qFLA1b* | 1 | 23,922,797~23,923,797 | All | sf0123923797 | 6.4E-10 | 13.1 |  |  |
| *qFLA1c* | 1 | 28,156,776~28,254,104 | All | sf0128253104 | 1.2E-10 | 6.9 |  |  |
| *qFLA1g* | 1 | 41,709,581~42,474,038 | All | sf0141889675 | 4.3E-07 | 15.4 |  |  |
| *qFLA2d* | 2 | 24,632,837~24,729,492 | All | sf0224673066 | 3.8E-09 | 1.9 |  |  |
| *qFLA3b* | 3 | 3,387,969~3,840,995 | All | sf0303533203 | 3.1E-10 | 4.6 | *PGL1*  *OsHLH153* |  |
| *qFLA3g* | 3 | 32,646,004~32,647,004 | All | sf0332647004 | 1.5E-12 | 5.4 |  |  |
| *qFLA4c* | 4 | 17,368,909~17,472,586 | All | sf0417448366 | 3.3E-09 | 2.6 |  |  |
| *qFLA4d* | 4 | 27,768,991~27,789,643 | All | sf0427789643 | 2.2E-09 | 0.2 |  |  |
| *qFLA4e* | 4 | 28,232,717~28,234,717 | All | sf0428233717 | 1.2E-08 | 2.2 |  |  |
| *qFLA6a* | 6 | 2,330,984~2,334,089 | All | sf0602331984 | 1.6E-09 | 0.4 |  |  |
| *qFLA6b* | 6 | 4,541,644~4,833,334 | All | sf0604700910 | 6.5E-09 | 0.8 |  | *QFla6* [23] |
| *qFLA6e* | 6 | 21,300,559~21,675,647 | All | sf0621376534 | 1.6E-09 | 1.0 |  |  |
| *qFLA8a* | 8 | 5,173,405~5,525,170 | All | sf0805323634 | 1.9E-10 | 2.1 |  |  |
| *qFLA8c* | 8 | 9,731,090~9,994,465 | All | sf0809856936 | 7.2E-09 | 3.3 |  |  |
| *qFLA8d* | 8 | 14,671,710~14,803,518 | All | sf0814734947 | 4.4E-09 | 0.8 |  |  |
| *qFLA8g* | 8 | 22,191,978~22,193,978 | All | sf0822192978 | 4.7E-10 | 1.0 |  |  |
| *qFLA9c* | 9 | 15,834,306~15,881,738 | All | sf0915858285 | 5.6E-11 | 0.9 |  | *fla9* [24] |
| *qFLA10c* | 10 | 13,189,967~13,744,203 | All | sf1013551393 | 1.0E-06 | 0.6 | *OsHLH174*  *OsHLH173* |  |
| *qFLA2c* | 2 | 22,104,788~22,853,601 | Ind | sf0222551637 | 3.3E-08 | 14.1 |  |  |
| *qFLA5a* | 5 | 9,571,194~9,813,283 | Ind | sf0509812283 | 4.6E-08 | 4.3 |  |  |
| *qFLA5b* | 5 | 28,206,223~28,775,275 | Ind | sf0528485157 | 5.4E-07 | 7.2 |  | *QFla5* [23] |
| *qFLA10a* | 10 | 7,768,445~7,814,593 | Ind | sf1007769062 | 4.6E-09 | 5.2 |  |  |
| *qFLA1c* | 1 | 28,156,776~28,254,104 | Jap | sf0128253104 | 1.5E-06 | 2.6 |  |  |
| *qFLA2e* | 2 | 24,789,662~24,797,944 | Jap | sf0224790662 | 1.7E-07 | 29.6 |  |  |
| *qFLA9d* | 9 | 16,296,311~16,715,109 | Jap | sf0916385175 | 1.3E-06 | 3.3 |  | *fla9* [24] |

a. The SNP ID is composed of three parts: sf, the number of chromosome and the genome position (MSU.V6), eg. sf0112429224 indicates the SNP located in 12,429,224bp on chromosome 1 (MSU.V6).

b. *PGL1*, *POSITIVE REGULATOR OF GRAIN LENGTH 1*, *LOC_Os03g07510*; *Os153*, *OsbHLH153*, *LOC_Os03g07540*; *Os174*, *OsbHLH174*, *LOC_Os10g26410*; *Os173*, *OsbHLH173*, *LOC_Os10g26460*.
