# Supplementary material for: Genome-wide association studies reveal that members of bHLH subfamily 16 share a conserved function in regulating flag leaf angle in rice (Oryza sativa)
Source: PLoS Genet. 2018 Apr 4;14(4):e1007323. doi: 10.1371/journal.pgen.1007323 (PMC5902044; doi:10.1371/journal.pgen.1007323)
Supplement: S2 Table — (DOCX) [file pgen.1007323.s002.docx]

**S2 Table Significant association loci for rice flag leaf angle only detected in Wuhan using the LMM**

| QTL ID | Chr | Local LD region (bp) | class | SNP ID ^a^ | P_LMM | Var % | known genes ^b^ | Known QTLs |
| --- | --- | --- | --- | --- | --- | --- | --- | --- |
| *qFLA1d* | 1 | 29,392,924~30,129,225 | All | sf0129476475 | 2.8E-12 | 19.3 | *OsBRI1* |  |
| *qFLA2a* | 2 | 2,158,922~2,645,417 | All | sf0202365270 | 9.9E-09 | 12.8 |  |  |
| *qFLA2f* | 2 | 31,360,841~31,541,252 | All | sf0231470445 | 3.0E-10 | 5.9 | *PGL2* | *QFla2* [23] |
| *qFLA3c* | 3 | 5,126,605~5,355,837 | All | sf0305249711 | 2.9E-09 | 3.9 |  |  |
| *qFLA3d* | 3 | 9,821,295~9,898,658 | All | sf0309829580 | 3.7E-10 | 3.0 |  | *qFLA3.2* [22] |
| *qFLA3f* | 3 | 28,171,853~28,657,302 | All | sf0328295584 | 7.3E-12 | 2.6 |  |  |
| *qFLA4a* | 4 | 1,089,784~1,330,103 | All | sf0401245161 | 8.5E-09 | 2.5 |  |  |
| *qFLA5b* | 5 | 28,206,223~28,775,275 | All | sf0528443224 | 1.4E-11 | 1.1 |  | *QFla5* [23] |
| *qFLA6c* | 6 | 5,479,047~5,481,047 | All | sf0605480047 | 1.6E-08 | 2.9 |  | *QFla6* [23] |
| *qFLA6f* | 6 | 22,302,956~22,545,957 | All | sf0622510255 | 1.4E-10 | 1.3 |  |  |
| *qFLA7b* | 7 | 4,587,362~4,743,465 | All | sf0704672463 | 1.3E-08 | 3.1 |  |  |
| *qFLA7e* | 7 | 28,893,769~29,673,928 | All | sf0728914351 | 6.2E-09 | 0.3 |  |  |
| *qFLA8b* | 8 | 8,972,704~9,060,591 | All | sf0808973704 | 9.0E-09 | 0.6 |  |  |
| *qFLA8e* | 8 | 16,444,758~16,800,442 | All | sf0816610009 | 1.3E-08 | 0.2 |  |  |
| *qFLA8h* | 8 | 22,932,602~22,975,868 | All | sf0822933602 | 5.6E-09 | 0.2 |  |  |
| *qFLA8i* | 8 | 26,144,917~26,619,563 | All | sf0826224010 | 8.5E-09 | 1.6 |  |  |
| *qFLA8j* | 8 | 27,965,589~28,101,784 | All | sf0828047813 | 2.1E-07 | 0.2 | *OsSPY* |  |
| *qFLA9a* | 9 | 851,490~865,444 | All | sf0900860607 | 9.7E-09 | 0.6 |  |  |
| *qFLA10b* | 10 | 11,472,188~11,580,579 | All | sf1011580488 | 7.5E-12 | 1.2 |  |  |
| *qFLA12c* | 12 | 15,481,370~15,579,807 | All | sf1215579807 | 3.0E-09 | 0.4 |  |  |
| *qFLA12d* | 12 | 17,334,511~17,558,740 | All | sf1217522965 | 1.4E-08 | 0.3 |  |  |
| *qFLA1f* | 1 | 41,336,043~41,612,137 | Ind | sf0141345784 | 4.4E-08 | 30.8 |  | *fla1* [24] |
| *qFLA1g* | 1 | 41,709,581~42,474,038 | Ind | sf0142057872 | 1.9E-08 | 5.5 |  |  |
| *qFLA2b* | 2 | 6,392,560~7,227,128 | Ind | sf0206393560 | 2.6E-08 | 1.3 |  |  |
| *qFLA4b* | 4 | 12,775,280~12,777,280 | Ind | sf0412776280 | 6.0E-08 | 1.7 |  |  |
| *qFLA6d* | 6 | 17,657,784~18,219,847 | Ind | sf0618121383 | 2.5E-09 | 2.2 |  | *QFla6* [23] |
| *qFLA7a* | 7 | 601,767~828,759 | Ind | sf0700792132 | 3.4E-08 | 1.1 |  |  |
| *qFLA7c* | 7 | 13,607,968~13,665,873 | Ind | sf0713665873 | 7.7E-08 | 6.2 |  |  |
| *qFLA7d* | 7 | 20,922,345~21,144,872 | Ind | sf0721006409 | 1.8E-08 | 0.1 |  | *QFla7* [23] |
| *qFLA11b* | 11 | 21,450,019~21,588,135 | Ind | sf1121563720 | 1.2E-08 | 0.7 |  |  |
| *qFLA12b* | 12 | 7,378,654~7,753,072 | Ind | sf1207493087 | 6.6E-08 | 10.7 |  |  |
| *qFLA12e* | 12 | 22,795,967~22,987,926 | Ind | sf1222942913 | 1.7E-09 | 0.3 |  |  |
| *qFLA8e* | 8 | 16,444,758~16,800,442 | Jap | sf0816541804 | 2.3E-06 | 10.7 |  |  |
| *qFLA9b* | 9 | 5,404,703~5,466,985 | Jap | sf0905465985 | 2.1E-09 | 3.0 |  |  |

a. The SNP ID is composed of three parts: sf, the number of chromosome and the genome position (MSU.V6), eg. sf0129476475 indicates the SNP located in 29,476,475bp on chromosome 1 (MSU.V6).

b. *OsBRI1*, *Oryza sativa BRASSINOSTEROID INSENSITIVE 1*, *LOC_Os01g52050*; *PGL2*, *POSITIVE REGULATOR OF GRAIN LENGTH 2*, *LOC_Os02g51320*; *OsSPY*, *Oryza sativa SPINDLY*, *LOC_Os08g44510*.
