# Supplementary material for: Genome-wide association studies reveal that members of bHLH subfamily 16 share a conserved function in regulating flag leaf angle in rice (Oryza sativa)
Source: PLoS Genet. 2018 Apr 4;14(4):e1007323. doi: 10.1371/journal.pgen.1007323 (PMC5902044; doi:10.1371/journal.pgen.1007323)
Supplement: S3 Table — (DOCX) [file pgen.1007323.s003.docx]

**S3 Table Haplotype-level association analysis of candidate genes using 529 accessions**

| Pop Gene | All | | | Ind | | | Jap | | |
| --- | --- | --- | --- | --- | --- | --- | --- | --- | --- |
|  | N | *P*_HN | *P*_WH | N | *P*_HN | *P*_WH | N | *P*_HN | *P*_WH |
| *OsBRI1* | 7 | 7.2E-35 | 1.26E-28 | 2 | 1.5E-05 | 0.0001 | 4 | 4.0E-06 | 0.0279 |
| *OsSPY* | 6 | 1.5E-30 | 2.1E-29 | 4 | 0.3351 | 0.3908 | 3 | 0.0003 | 0.0005 |
| *PGL1* | 10 | 2.0E-26 | 5.1E-21 | 7 | 0.8433 | 0.5996 | 4 | 1.0E-06 | 0.0032 |
| *PGL2** | 7 | 2.2E-22 | 6.5E-21 | 6 | 0.3342 | 0.7331 | 2 | 0.0147 | 0.0572 |
| *OsbHLH153** | 8 | 2.1E-29 | 1.0E-18 | 6 | 0.3029 | 0.3683 | 4 | 8.2E-07 | 0.1243 |
| *OsbHLH173** | 7 | 1.4E-26 | 1.6E-25 | 4 | 0.8322 | 0.3442 | 4 | 0.0044 | 0.0094 |
| *OsbHLH174** | 11 | 1.1E-35 | 5.7E-29 | 5 | 0.4219 | 0.2476 | 8 | 9.5E-07 | 0.0047 |
| *ILI1** | 5 | 2.4E-25 | 2.0E-16 | 4 | 0.4513 | 0.5935 | 5 | 4.2E-06 | 0.0571 |
| *BU1** | 6 | 8.9E-05 | 1.0 E-4 | 6 | 0.4129 | 0.2841 | 2 | 0.4434 | 0.3234 |

* Haplotypes of these genes were constructed using the SNPs in the 2-kb promoter region and coding sequence region. For the other three, only SNPs in the gene body were included. N, the number of haplotypes in whole population and two major subpopulations (*indica* and *japonica*) using 529 accessions. HN, Hainan; WH, Wuhan. *P* value is the testing statistical of analysis of variance.
