# Supplementary material for: Genome-wide association studies reveal that members of bHLH subfamily 16 share a conserved function in regulating flag leaf angle in rice (Oryza sativa)
Source: PLoS Genet. 2018 Apr 4;14(4):e1007323. doi: 10.1371/journal.pgen.1007323 (PMC5902044; doi:10.1371/journal.pgen.1007323)
Supplement: S5 Table — (DOCX) [file pgen.1007323.s005.docx]

**S5 Table Primers used in this study.**

| Name | Primer sequence (5'-3') |
| --- | --- |
| Primers for RT-PCR |  |
| *Actin* |  |
| L | TGCTATGTACGTCGCCATCCAG |
| R | AATGAGTAACCACGCTCCGTCA |
| *Style2.1* |  |
| L | CAACTTCTACCGGAGATTCGC |
| R | ACTCCTGATGATTGCTGCTTCT |
| Primers for qRT-PCR analysis |  |
| *UBQ* |  |
| L | AACCAGCTGAGGCCCAAGA |
| R | ACGATTGATTTAACCAGTCCATGA |
| *Os153* |  |
| L | ACGAGCTCATCTCCAAGCTTCA |
| R | ATGTAGCTGCACGTCTCCTTGA |
| *Os173* |  |
| L | AGGAGACGTGCAGCTACATCC |
| R | ATGAGCAGGCCGCGGATGA |
| *Os174* |  |
| L | AAGCTGCTGAAGGAGATGTGC |
| R | TGAGGGCTGTTGCTGTCCAT |
| Primers for transgenic plants genotyping |  |
| *GUS* |  |
| L | CCAGGCAGTTTTAACGATCAGTTCGC |
| R | GAGTGAAGATCCCTTTCTTGTTACCG |
| *Style2.1* |  |
| L | CCTTACCACAACTTCCTCTCC |
| R | TGGCCTAGCTATAGTGACCAGC |
| Primers for vector construction |  |
| *Os153* |  |
| L | AGCGGTACCTAGTCGAGCCGCCGTGGTGGTGGT |
| R | AGCGGATCCCCGGAGGAGGCTCCGGATGATCTC |
| *Os173* |  |
| L | AGCGGTACCATGTCCAGCCGGAGCAGGTCGA |
| R | AGCGGATCCCATGAGCAGGCCGCGGATGAC |
| *Os174* |  |
| L | AGCGGTACCATGTCGGGGCGGCGAGCAT |
| R | AGCGGATCCGCGGAGGAGGCTGCGGATGA |
| *Style2.1* |  |
| L | GGTACCCCTTACCACAACTTCCTCTCC |
| R | GTCGACTGGCCTAGCTATAGTGACCAGC |
