# Supplementary figures and images for: Genome-wide association studies reveal that members of bHLH subfamily 16 share a conserved function in regulating flag leaf angle in rice (Oryza sativa)
Source: PLoS Genet. 2018 Apr 4;14(4):e1007323. doi: 10.1371/journal.pgen.1007323 (PMC5902044; doi:10.1371/journal.pgen.1007323)

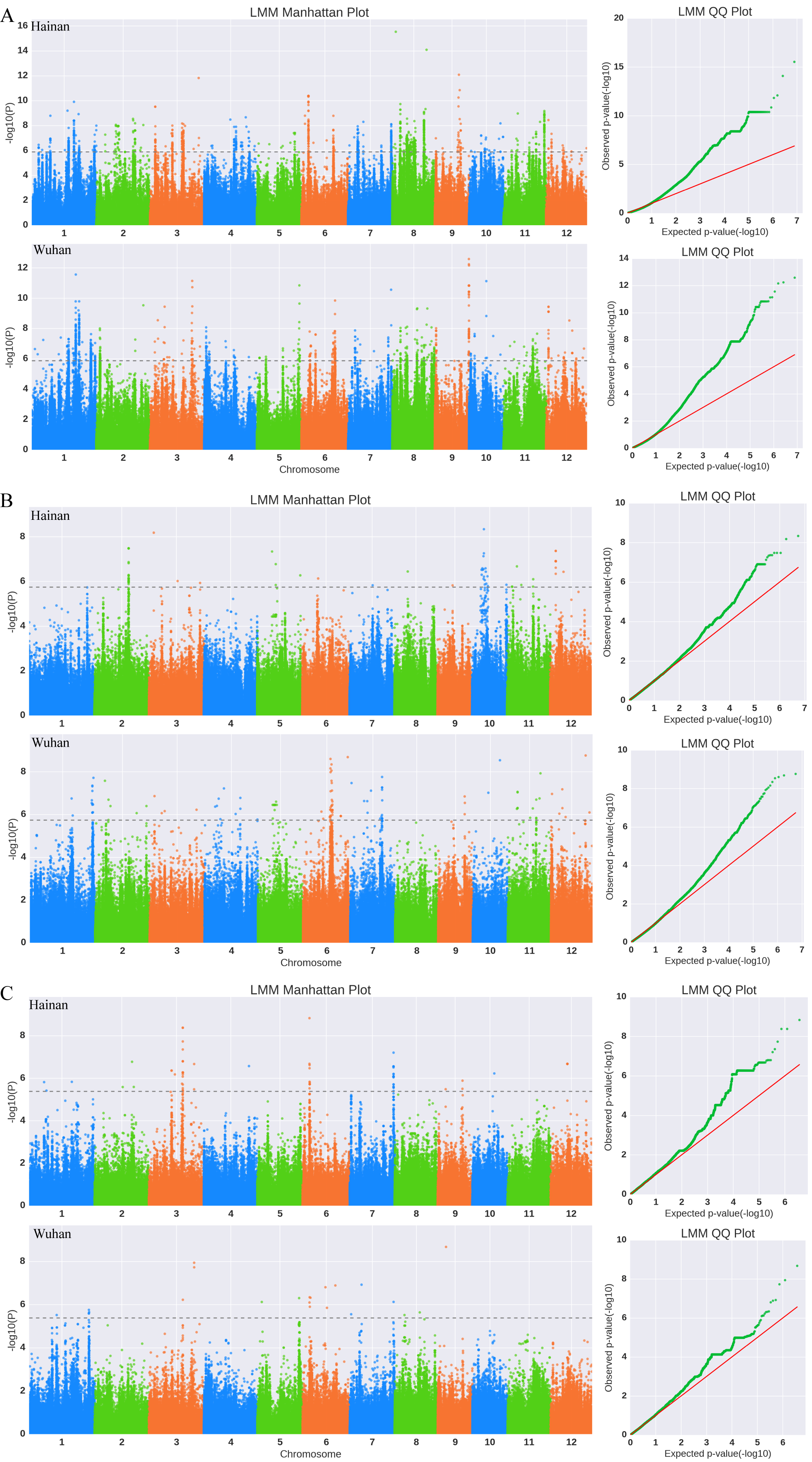

Supplement: S1 Fig — Manhattan plots and quantile-quantile plots for flag leaf angle in the full population (A), the indica subpopulation (B) and japonica subpopulation (C). The horizontal dashed lines of the Manhattan plots indicate the significance thresholds that are defined in the section of materials and methods. Lambda of quantile-quantile plots represents the expected null distribution and the observed P value. (TIF) [file pgen.1007323.s007.tif]

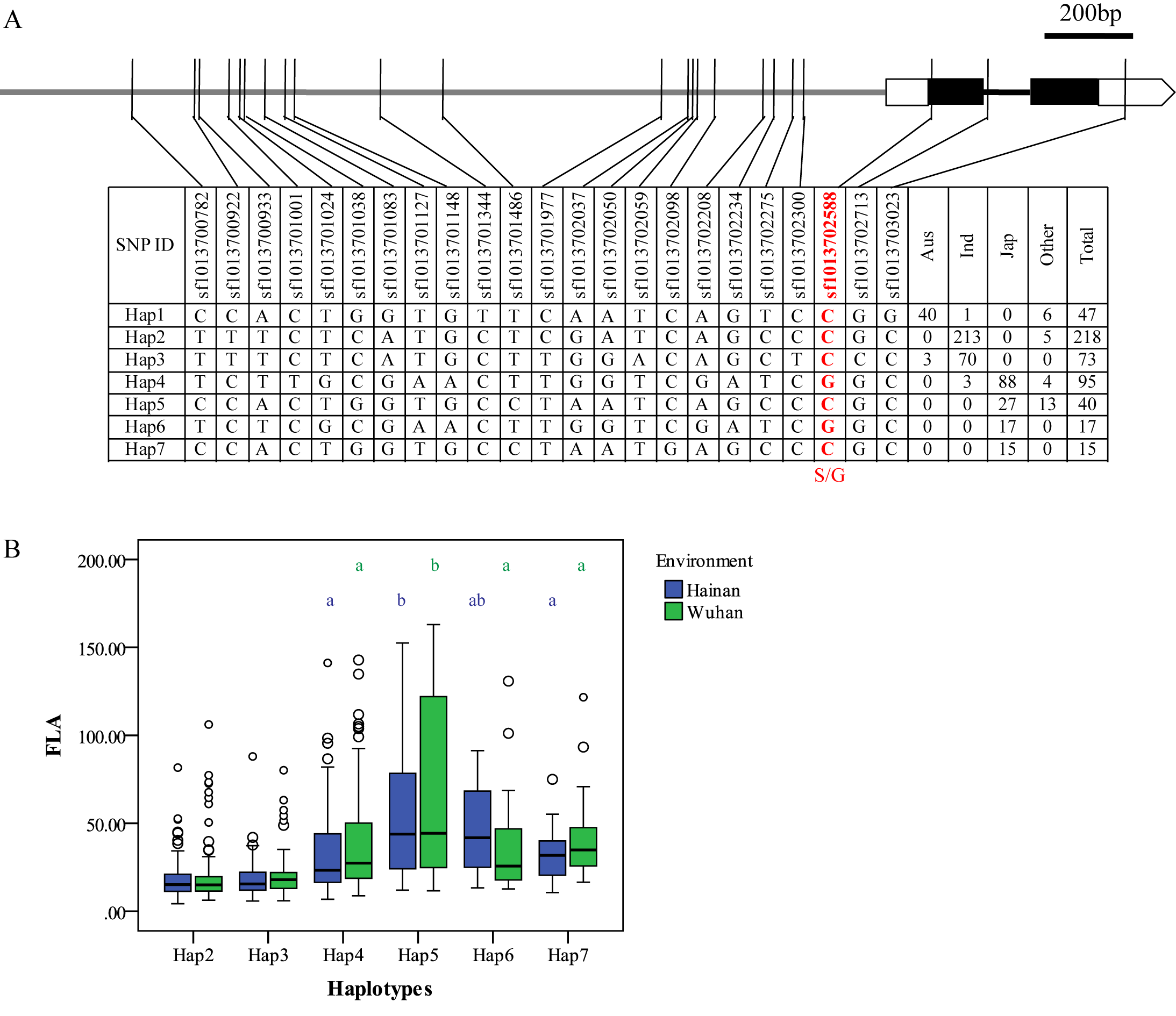

Supplement: S2 Fig — (A) Major haplotyes (haplotypes with more than 5accessions) of OsbHLH173 in the full population according to SNPs data from RiceVarMap version 1. The region contains 2-kb upstream and coding region. The SNP in red and bold is a non-Synonymous SNP. (B) Comparison of the FLA between Hap2 and Hap3 in indica rice and the FLA among Hap4-Hap7 in japonica rice using an independent t-test and a Duncan’s test (P< 0.05), respectively. (TIF) [file pgen.1007323.s008.tif]

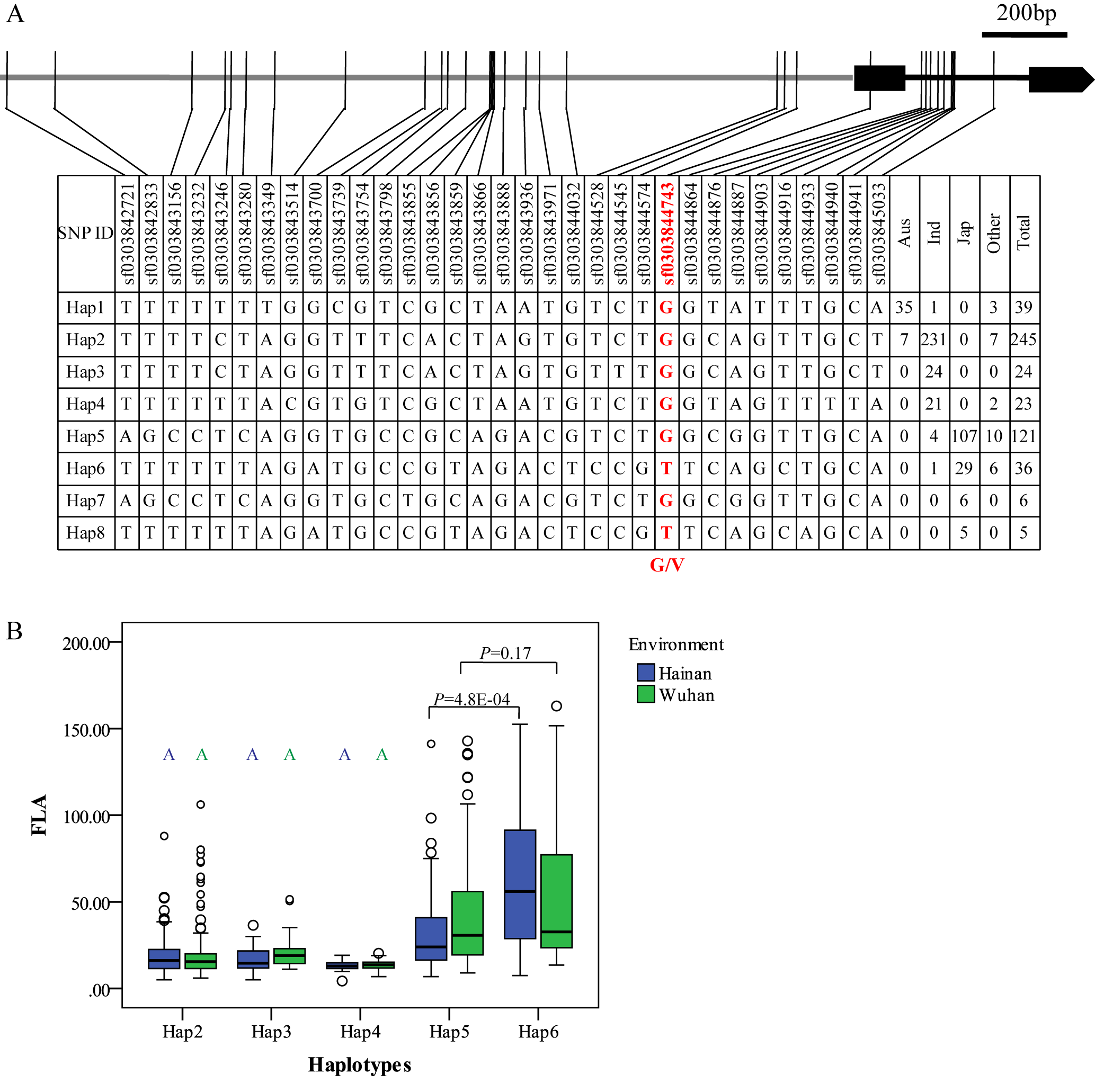

Supplement: S3 Fig — (A) Major haplotyes (haplotypes with more than 5accessions) of OsbHLH153 in the full population according to SNPs data from RiceVarMap version 1. The region contains 2 kb upstream and coding region. The SNP in red and bold is a non-Synonymous SNP. (B) Comparison of the FLA among Hap2-Hap4 in indica rice and the FLA between Hap5 and Hap6 in japonica rice using a Duncan’s test (P< 0.01) and an independent t-test, respectively. (TIF) [file pgen.1007323.s009.tif]

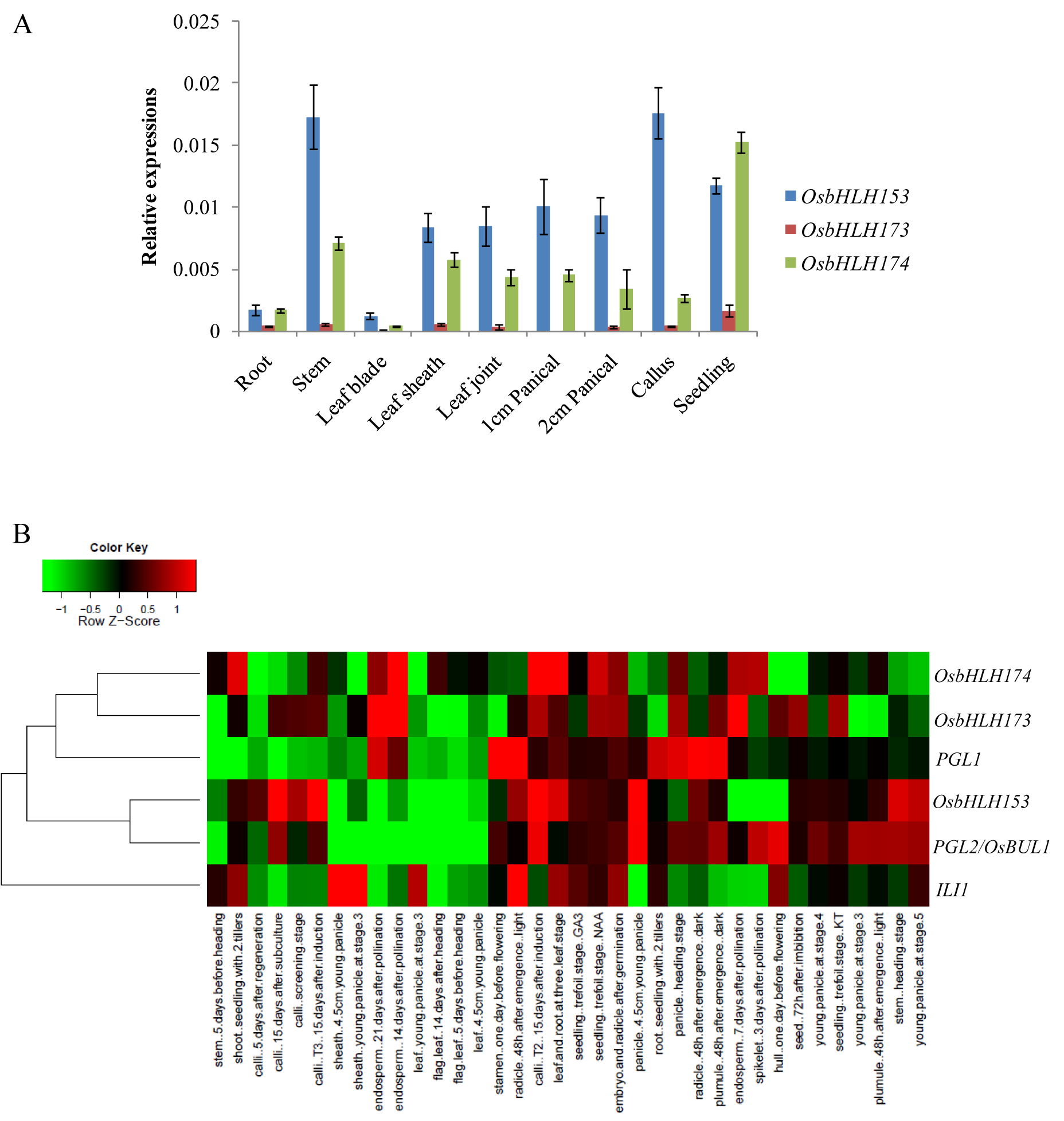

Supplement: S4 Fig — (A) qRT-PCR expression analysis of OsbHLH153, OsbHLH173 and OsbHLH174 in different tissues of wild type rice. The samples of Root, Stem, Leaf blade, Leaf sheath and Leaf joint were harvested at the transition period of vegetative growth to reproductive growth. (B) The expression profiling of subfamily 16 of bHLH in different tissues, and the expression data was extracted from a database (http://crep.ncpgr.cn/crep-cgi/home.pl). The expression data of BU1 was missing. (TIF) [file pgen.1007323.s010.tif]

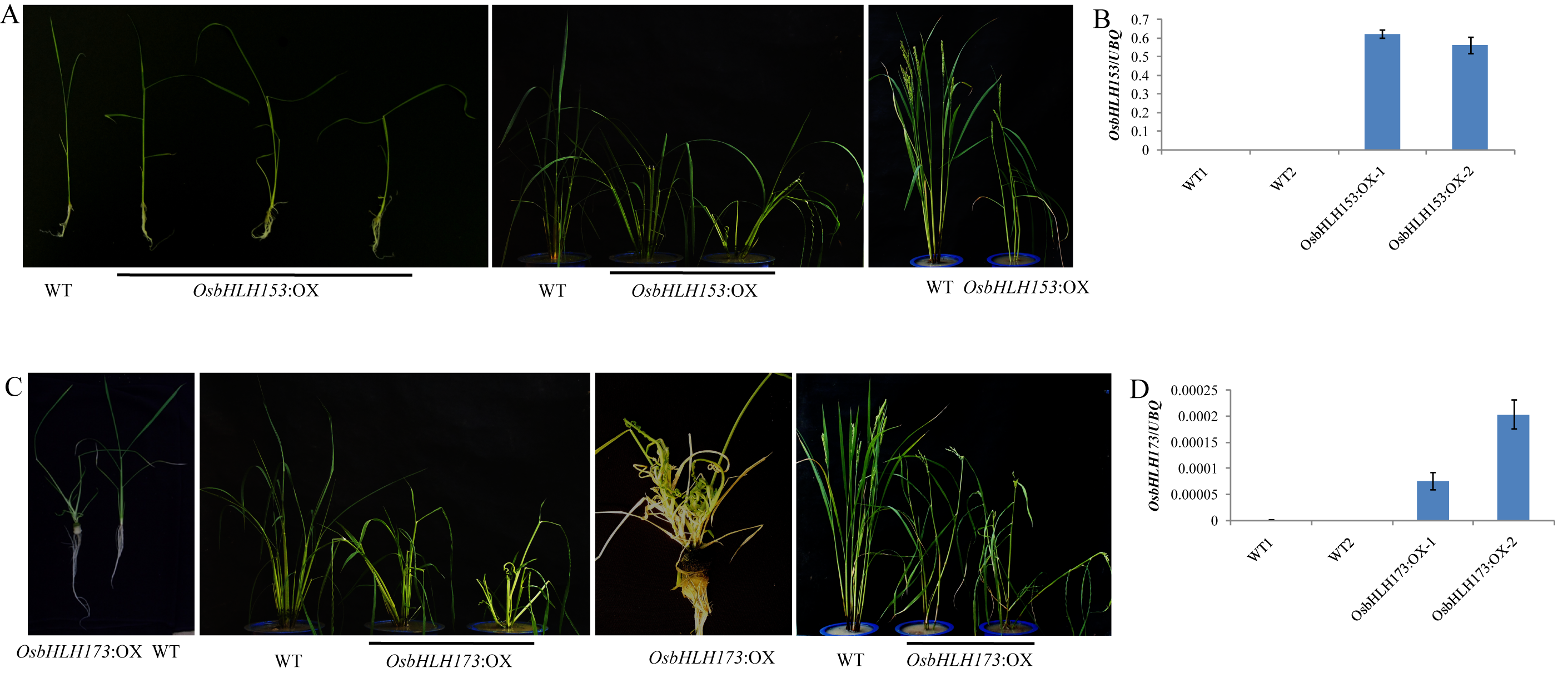

Supplement: S5 Fig — Overexpressing OsbHLH153 (A) and OsbHLH173 (C) transgenic plants increased leaf angle; (B) qRT-PCR analysis of the OsbHLH153 in wild type (WT) and OsbHLH153: OX using leaves at seedling stage; (D) qRT-PCR analysis of the OsbHLH173 in WT and OsbHLH173: OX using leaves at seedling stage. (TIF) [file pgen.1007323.s011.tif]

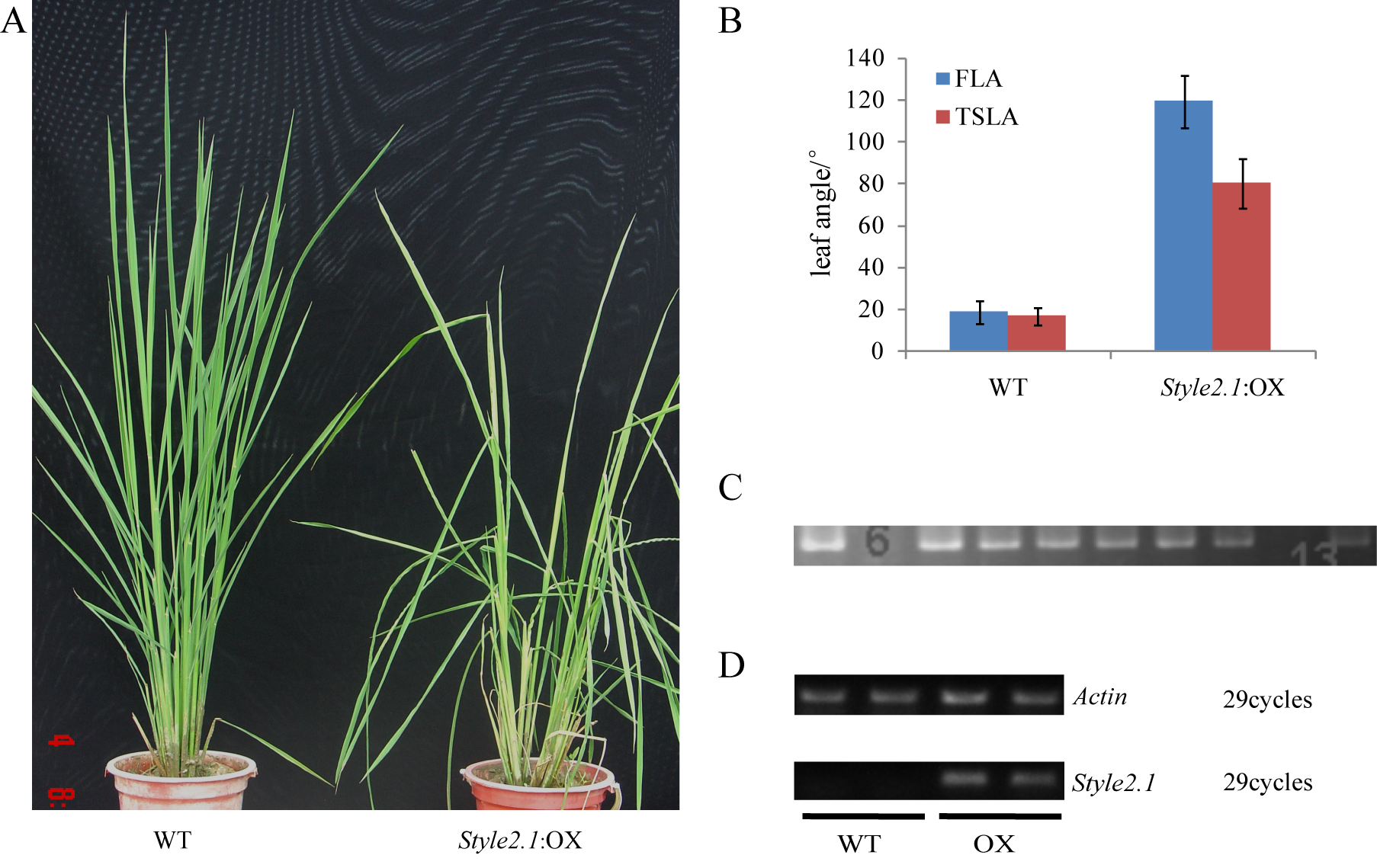

Supplement: S6 Fig — (A) The plant status of WT and Style2.1: OX at tillering stage; (B) FLA and TSLA of the wild type and Style2.1: OX, n≥ 5; (C) genotyping of transgenic plants Style2.1: OX; (D) reverse transcription PCR (RT-PCR) analysis of the transcripts of Style2.1 in WT and Style2.1: OX at seedling stage. (TIF) [file pgen.1007323.s012.tif]

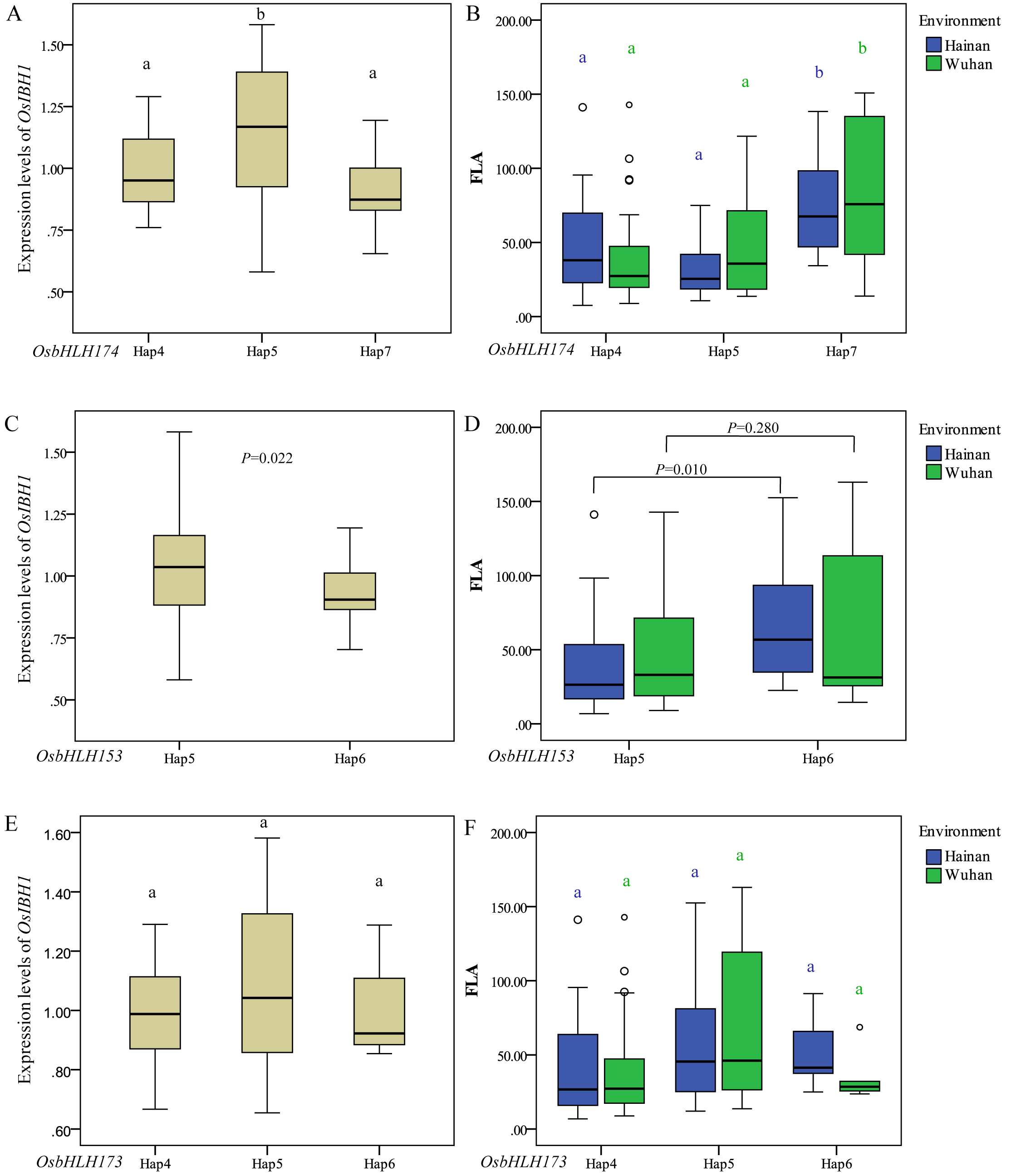

Supplement: S7 Fig — Comparison of the expression level of OsIBH1 among/between the haplotypes of OsbHLH174 (A), OsbHLH153 (C) and OsbHLH173 (E) in japonica using RNA-seq data by a Ducan’s test (P< 0.05). Comparison of FLA among/between the haplotypes of OsbHLH174 (B), OsbHLH153 (D) and OsbHLH173 (F) in the corresponding japonica accessions by a Ducan’s test (P< 0.05). (TIF) [file pgen.1007323.s013.tif]
